# Multi-site Neurogenin3 Phosphorylation Controls Pancreatic Endocrine Differentiation

**DOI:** 10.1016/j.devcel.2017.04.004

**Published:** 2017-05-08

**Authors:** Roberta Azzarelli, Christopher Hurley, Magdalena K. Sznurkowska, Steffen Rulands, Laura Hardwick, Ivonne Gamper, Fahad Ali, Laura McCracken, Christopher Hindley, Fiona McDuff, Sonia Nestorowa, Richard Kemp, Kenneth Jones, Berthold Göttgens, Meritxell Huch, Gerard Evan, Benjamin D. Simons, Douglas Winton, Anna Philpott

**Affiliations:** 1 Department of Oncology, University of Cambridge, Hutchison/MRC Research Centre, Hills Road, Cambridge CB2 0XZ, UK; 2 Wellcome Trust-Medical Research Council Cambridge Stem Cell Institute, University of Cambridge, Tennis Court Road, Cambridge CB2 1QR, UK; 3 Cancer Research UK Cambridge Research Institute, Li Ka Shing Centre, Robinson Way, Cambridge CB2 0RE, UK; 4 Max Planck Institute for the Physics of Complex Systems, Nöthnitzer Straße 38, 01187 Dresden, Germany; 5 Center for Systems Biology Dresden, Pfotenhauer Straße 108, 01307 Dresden, Germany; 6 Department of Biochemistry, University of Cambridge, Cambridge CB2 1GA, UK; 7 The Wellcome Trust/Cancer Research UK Gurdon Institute, University of Cambridge, Cambridge CB2 1QN, UK; 8 Cavendish Laboratory, Department of Physics, University of Cambridge, Cambridge CB3 0HE, UK; 9 Department of Haematology, Cambridge Institute for Medical Research, Hills Road, Cambridge CB2 0XY, UK

**Keywords:** proneural bHLH transcription factors, endocrine differentiation, pancreatic development, β cells, neurogenin3, pancreatic organoids, insulinoma, diabetes

## Abstract

The proneural transcription factor Neurogenin3 (Ngn3) plays a critical role in pancreatic endocrine cell differentiation, although regulation of Ngn3 protein is largely unexplored. Here we demonstrate that Ngn3 protein undergoes cyclin-dependent kinase (Cdk)-mediated phosphorylation on multiple serine-proline sites. Replacing wild-type protein with a phosphomutant form of Ngn3 increases α cell generation, the earliest endocrine cell type to be formed in the developing pancreas. Moreover, un(der)phosphorylated Ngn3 maintains insulin expression in adult β cells in the presence of elevated c-Myc and enhances endocrine specification during ductal reprogramming. Mechanistically, preventing multi-site phosphorylation enhances both Ngn3 stability and DNA binding, promoting the increased expression of target genes that drive differentiation. Therefore, multi-site phosphorylation of Ngn3 controls its ability to promote pancreatic endocrine differentiation and to maintain β cell function in the presence of pro-proliferation cues and could be manipulated to promote and maintain endocrine differentiation in vitro and in vivo.

## Highlights

• Ngn3 can be phosphorylated on multiple serine-proline sites
• Un(der)phosphorylated Ngn3 is more stable and active than wild-type Ngn3
• Ngn3 phosphorylation regulates pancreatic endocrine cell generation in vivo
• Un(der)phosphorylated Ngn3 promotes endocrine cell reprogramming in organoids

## Graphical Abstract

**Figure:**
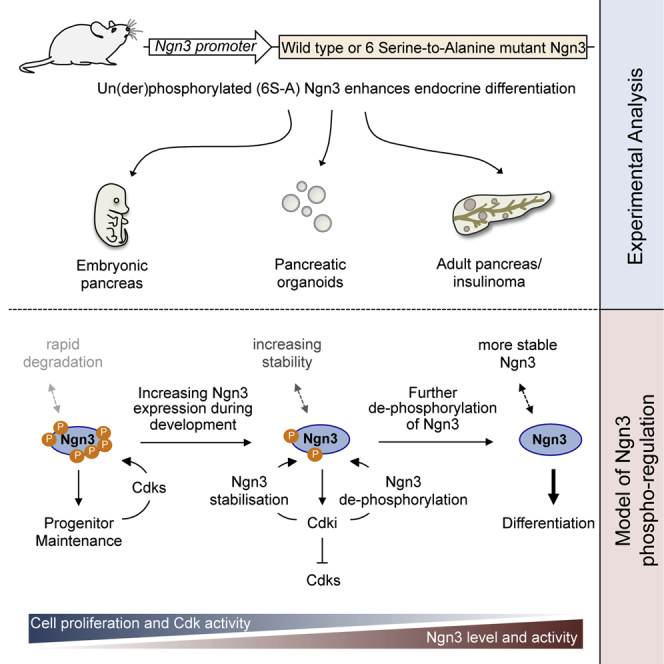

## Teaser

Azzarelli et al. show that multi-site phosphorylation of Neurogenin3 regulates pancreatic endocrine differentiation during development, and maintenance of adult β cell function in the presence of pathological pro-proliferative cues. The results suggest that dephosphorylation of Neurogenin3 may improve β cell generation in vitro and help maintain islet function in disease.

## Introduction

While endocrine cells represent only 1%–5% of the entire pancreas, they play a crucial role in physiological processes including glucose homeostasis; loss or dysfunction of β cells can lead to diabetes. In vitro differentiation and transplantation of bona-fide functional β cells can regulate blood sugar in diabetic mouse models (Pagliuca et al., 2014, Rezania et al., 2014), while β cell protection or replacement in diabetic patients may improve long-term glycemic control. Moreover, a fasting and refeeding regime may lead to β cell regeneration, with improvement of blood sugar regulation (Cheng et al., 2017). However, further improvements in β cell replacement or regeneration require a comprehensive understanding of the generation and maintenance of pancreatic endocrine cells in development and adulthood.

During pancreatic development, endocrine cells originate from bipotent ductal-endocrine progenitors in the embryonic ductal epithelium that become specified to the endocrine lineage upon expression of the pro-endocrine basic-helix-loop-helix (bHLH) transcription factor Neurogenin3 (Ngn3 or Neurog3). Overexpression of Ngn3 from the early pancreatic Pdx1 promoter induces early and ectopic differentiation of islet cells (Schwitzgebel et al., 2000), while controlled Ngn3 overexpression at defined developmental stages results in sequential formation of the different endocrine cell types (Johansson et al., 2007, Schwitzgebel et al., 2000). Furthermore, Ngn3 is a central component of transcription factor reprogramming cocktails that can *trans*-differentiate adult exocrine acinar cells into functional β cells (Zhou et al., 2008), while cytokine-induced in vivo reprogramming of acinar cells into β cells also requires the transition through a Ngn3-positive stage, indicative of reversion back to a progenitor-like phase that re-instates developmental programs of endocrine differentiation (Baeyens et al., 2014). Ngn3 is also required for adult β cell function (Wang et al., 2009).

While in adult pancreatic homeostasis new β cells are generated by proliferation of pre-existing β cells (Dor et al., 2004), β cell regeneration upon specific types of injury and genetic manipulation may require the reactivation of a population of facultative stem cells expressing elevated functional Ngn3 (Al-Hasani et al., 2013, Van de Casteele et al., 2013, Xu et al., 2008). Ductal cells retain the capacity to differentiate to endocrine islet cells in vitro after ectopic expression of Ngn3 (Heremans et al., 2002), in particular when co-expressed with other transcription factors (Lee et al., 2013). Moreover, pancreatic ductal cells can be expanded in vitro as 3D organoids for an extensive period of time, yet maintain the capacity to differentiate toward the endocrine lineage; a process that requires the passage through an Ngn3-positive phase (Huch et al., 2013). Overall, Ngn3 clearly plays a crucial role in endocrine cell generation, regeneration, and reprogramming, yet its regulation at the level of Ngn3 protein has barely been investigated (Roark et al., 2012, Sancho et al., 2014).

Focusing here on its post-translational modification, we show that Ngn3 is phosphorylated on multiple serine-proline (SP) sites by cyclin-dependent kinases (Cdks). A phosphomutant form of Ngn3 expressed in place of the wild-type (WT) protein results in perturbed regulation of endocrine cells in vivo. Mechanistically, we see that this phosphomutant Ngn3 is more stable than the WT protein and also shows increased binding at key downstream target genes. Compared with the WT protein, phosphomutant Ngn3 enhances expression of endocrine target genes in reprogramed ductal cells, while also maintaining insulin expression and blood glucose homeostasis in the presence of a c-Myc driven pro-proliferative stimulus in vivo. We conclude that multi-site phosphorylation of Ngn3 controls its ability to promote and maintain endocrine differentiation in the embryonic and adult pancreas.

## Results

### Ngn3 Is Phosphorylated on Multiple Sites by Cdks

**Figure 1:**
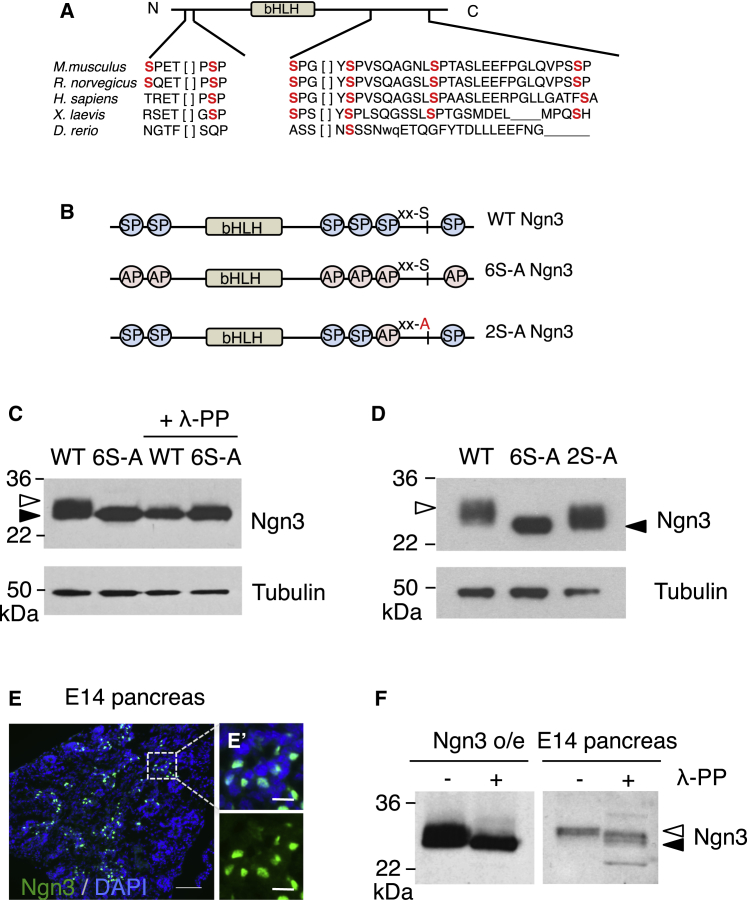
Ngn3 Is Phosphorylated on Multiple Sites (A) Six serine-proline (SP) sites in mouse Ngn3, showing conservation across species. (B) Schematic representation of the SP sites mutated in 6S-A Ngn3 and 2S-A Ngn3. (C) Western blot showing that Ngn3 is phosphorylated in mammalian HEK cells; treatment with and without phosphatase λ (λ-PP) is indicated. (D) Western blot showing that 6S-A and 2S-A Ngn3 are phosphorylated in insulinoma-derived MIN6 cells, with tubulin as a loading control. (E) Ngn3 immunostaining in E14.5 mouse embryonic pancreas; nuclei are counterstained with DAPI (blue). Scale bars, 50 μm (E) and 10 μm (E’). (F) Ngn3 expression and phosphorylation in HEK cells overexpressing Ngn3 (Ngn3 o/e) compared with E14.5 murine embryonic pancreas; λ-PP, phosphatase λ. Solid and open arrowheads in (C, D, and F) indicate un(der)phosphorylated and phosphorylated Ngn3, respectively. See also Figure S1.

Ngn3 is closely related to other bHLH transcription factors, whose activity is regulated by phosphorylation on SP sites, e.g., Ngn2, Ascl1, Atoh1, and Olig2 (Ali et al., 2011, Ali et al., 2014, Forget et al., 2014). Ngn3 contains six SP sites that can potentially be targeted by proline-directed kinases such as Cdks, MAPKs, and ERKs, and five of these sites are conserved across species (Figure 1A). To investigate phosphorylation on SP sites, we expressed WT Ngn3 or 6S-A Ngn3 (where all six SP sites had been mutated to alanine-proline) (Figure 1B) in HEK cells, then compared migration on SDS-PAGE. Ngn3 phosphorylation is demonstrated by slower migration of WT compared with 6S-A Ngn3, which is enhanced by phosphatase λ (λ-PP) treatment (Figure 1C). WT Ngn3 also migrates more slowly than 6S-A Ngn3 in MIN6 cells, derived from the islet cancer insulinoma (Ishihara et al., 1993), as well as in mPAC cells derived from pancreatic ductal adenocarcinoma (Yoshida and Hanahan, 1994) (Figure 1D). As reported previously (De Vas et al., 2015, Gradwohl et al., 2000, Smith et al., 2010, Zhou et al., 2007), Ngn3 is highly expressed in the developing embryonic pancreas in scattered endocrine precursor cells (Figures 1E, E’, and S1A). Co-staining with an epithelial marker at embryonic day 16 (E16) shows that Ngn3 is expressed in cells of the ductal epithelium prior to delamination (Figures S1B and S1B’) (Rukstalis and Habener, 2007). Endogenous Ngn3 from E14 embryonic pancreas also shows SDS-PAGE retardation compared with samples treated with phosphatase (Figures 1F and S1C), demonstrating phosphorylation of endogenously expressed Ngn3 protein.

Phosphorylation of Ngn3 on two C-terminal serines, one of which is an SP site, has previously been reported to regulate Ngn3 protein stability (Sancho et al., 2014). To determine whether the phosphorylation of Ngn3 we observe is solely due to phosphorylation on these two sites (mutated in 2S-A Ngn3, Figures 1B and 1D), we compared SDS-PAGE migration of WT Ngn3, 6S-A Ngn3, and a 2S-A Ngn3. 2S-A Ngn3 migrates faster than WT Ngn3 (Figure 1D, open arrowhead), but more slowly than 6S-A Ngn3 (Figure 1D, black arrowhead), indicating phosphorylation on more than two serines.

**Figure 2:**
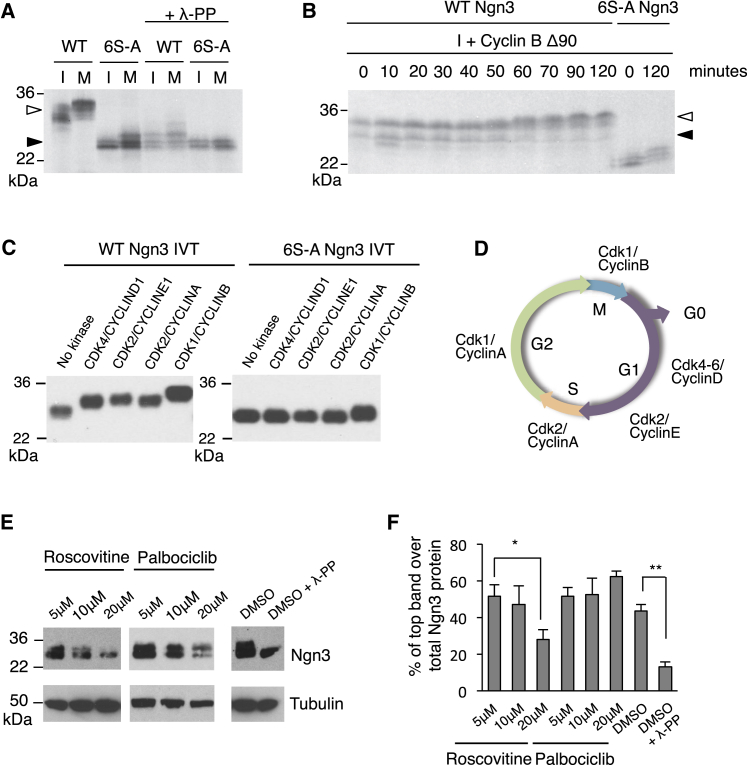
Ngn3 Is Phosphorylated by Cyclin-Dependent Kinases (A) SDS-PAGE separation of in vitro translated (IVT) radiolabeled WT Ngn3 or 6S-A Ngn3 incubated in interphase (I) or mitotic (M) *Xenopus* extracts, treated with phosphatase λ (λ-PP), as indicated, or (B) incubated in I extract plus cyclin B Δ90; samples removed at increasing times. Solid and open arrowheads indicate un(der)phosphorylated and phosphorylated Ngn3, respectively. (C) In vitro kinase assay showing IVT WT and 6S-A Ngn3 proteins after incubation with human recombinant CYCLIN/CDKs, as labeled. (D) Schematic of the activity of cyclin/Cdks in the different phases of cell cycle. (E) Western blot of HA-tagged Ngn3 expressed in ductal mPAC cells after treatment with Cdk inhibitors, treatment of Ngn3 with λ-PP as a positive control for protein dephosphorylation, tubulin as loading control. (F) Graphs showing the relative amount of the slowest migrating band of phosphorylated Ngn3, compared with the total amount of Ngn3 protein. n = 3 independent experiments, a representative blot is shown. Mean ± SEM. Student's t test, ^∗^p < 0.05, ^∗∗^p < 0.01.

Ngn2 and Ascl1, homologs of Ngn3, are phosphorylated by Cdks in a cell-cycle-dependent manner on multiple sites in the N and C termini (Ali et al., 2011, Ali et al., 2014, Forget et al., 2014) (Figure 2D). To investigate whether Ngn3 phosphorylation is affected by changing levels of Cdks, we turned to *Xenopus* egg extracts that recapitulate an interphase (I) or mitotic (M) environment (Figure 2A) and have long been used to investigate Cdk-dependent phosphorylation (Philpott and Yew, 2008). Compared with phosphomutant 6S-A Ngn3, WT Ngn3 migration on SDS-PAGE is slowed in I and even more so in M extract, a retardation reversed by phosphatase treatment (Figure 2A). Addition of non-degradable cyclin B to I extract directly activates Cdk1 and induces its entry into M phase after 30–40 min. This is paralleled by progressive retardation of WT Ngn3 migration (Figure 2B).

*Xenopus* I egg extracts have active cyclin E/Cdk2, while addition of non-degradable cyclin B will activate Cdk1 as extracts enter mitosis. However, cyclin D/Cdk4 is not present in eggs (Philpott and Yew, 2008). To determine which Cdks are capable of phosphorylating Ngn3, we undertook in vitro kinase assays using human recombinant Cdk/cyclin pairs. Slowed migration on SDS-PAGE reveals that Ngn3 can be phosphorylated by all the Cdks tested, but to differing extents. Retardation of SDS-PAGE migration indicates that Cdk1 is the most potent kinase for Ngn3, supporting our findings in *Xenopus* egg extracts (Figure 2C), while Cdk4 phosphorylation results in the smallest migration change (Figure 2C). 6S-A Ngn3 migration is unaffected by incubation with Cdk2 or Cdk4, indicating that these kinases phosphorylate on SP sites (Figure 2C). A small retardation of 6S-A Ngn3 is observed with Cdk1, as well as after incubation in M extract (Figures 2B and 2C); we note that 6S-A Ngn3 has one threonine-proline site that remains a potential target site for Cdk1.

To further explore the identity of Cdks phosphorylating Ngn3 in mammalian cells, we treated Ngn3-expressing cells with Roscovitine, an inhibitor with selectivity for Cdk1/2 (and 5), alongside Palbociclib, an inhibitor of Cdk4/6 (Asghar et al., 2015, Meijer and Kim, 1997). Only the faster migrating form of Ngn3 remained after Roscovitine treatment, while the Ngn3 doublet was clearly still visible in Palbociclib (Figure 2E). We noted that Roscovtitine and Palbociclib suppressed overall Ngn3 levels, consistent with off-target effects suppressing the transcriptional Cdks, Cdk7, and Cdk9 (Asghar et al., 2015). Therefore, to mitigate against any effects of loss of overall Ngn3 protein, we quantitatively compared the amount of the slower-migrating form of Ngn3 with total Ngn3 protein in three independent experiments, with and without kinase inhibitors (Figures 2E and 2F). Roscovitine treatment resulted in a relative accumulation of faster-migrating un(der)phosphorylated Ngn3 forms, while Palbociclib has no detectable effect on Ngn3 phosphorylation (Figures 2E and 2F).

Thus, we see that Ngn3 is directly phosphorylated by Cdks, and in particular Cdk1 and Cdk2. Ngn3 can be phosphorylated by high levels of Cdk4 in vitro, but failure to observe Ngn3 dephosphorylation in response to Palbociclib indicates that Cdk4 is not a major kinase for Ngn3 in mPAC cells. Instead, our evidence is consistent with a more prominent role for Cdk1 and Cdk2 compared with Cdk4 in the phosphoregulation of Ngn3 in pancreatic cells. We next investigated the functional consequences of preventing Cdk-dependent Ngn3 phosphorylation during pancreas formation.

### Ngn3 Phosphorylation Controls the Number of Endocrine Cells in the Embryonic Pancreas

**Figure 3:**
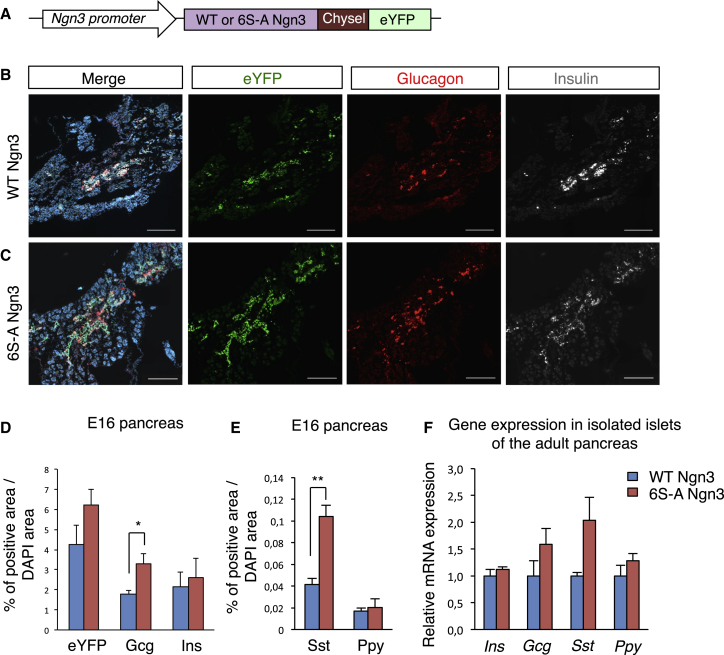
6S-A Ngn3 Enhances α and δ Cells in the Embryonic Pancreas (A) WT and 6S-A Ngn3 mouse model schematic. (B and C) Immunohistochemistry for eYFP (green), glucagon (red), and insulin (gray) in E16 embryonic pancreas from WT (B) and 6S-A (C) Ngn3 animals, nuclei counterstained with DAPI (blue). Scale bar, 200 μm. (D and E) Quantification of the percentage of areas that are eYFP+, insulin+, glucagon+ (D), somatostatin+ (Sst), and pancreatic polypeptide Y+ (Ppy) (E), in E16 embryonic pancreas. n = 4 mean ± SEM. Student's t test, ^∗^p < 0.05, ^∗∗^p < 0.01. (F) Relative hormone gene expression in adult pancreatic islets by qPCR, normalized to EF-1α expression. n = 3 mean ± SEM. See also Figures S2–S4.

Ngn3 plays a major role in endocrine specification and differentiation during development (Gradwohl et al., 2000, Rukstalis and Habener, 2009). To determine whether phosphorylation status of Ngn3, expressed at the normal time and at endogenous levels, can influence endocrine cell fate, we generated a knockin mouse that carries 6S-A Ngn3 separated from eYFP by 2A peptide, and transcribed homozygously from the Ngn3 locus, with a matched control WT Ngn3 eYFP mouse line (Figures 3A and S2A). As expected, in the embryonic pancreas, 6SA Ngn3 is dephosphorylated, running as a single, faster-migrating form compared with the WT protein (Figure S2B). To determine the developmental effects of preventing phosphorylation of Ngn3, we then quantified the relative amount of the distinct endocrine cell types in WT Ngn3 and 6S-A Ngn3 mice (percentage of hormone-positive area normalized to total DAPI area) at embryonic stage E16 (Johansson et al., 2007, Rukstalis and Habener, 2009), when endocrine cells will be largely specified (Figures 3B–3E, S3A, and S3B). Glucagon-positive cells are the first cell type to arise during pancreatic endocrine differentiation and numbers approximately double in 6S-A Ngn3 animals compared with controls (3.3% ± 0.5% versus 1.8% ± 0.2%, n = 4) (Figure 3D). Insulin or Ppy-positive cells are very similar in WT and 6S-A Ngn3 animals. Somatostatin (Sst)-positive cells also significantly increase (0.1% ± 0.01% in 6S-A Ngn3 compared with 0.04% ± 0.006% in Ngn3 WT, n = 3) (Figure 3E). We also observed a trend toward more eYFP+ cells (marking Ngn3 expression) in 6S-A embryonic pancreata (Figures 3B–3D), which did not reach statistical significance and this result was consistent with our quantification of Ngn3 protein staining (Figures S3C–S3E).

We then investigated the composition of the adult endocrine compartment in 6S-A Ngn3 mice, when Ngn3 levels have substantially dropped (Rukstalis and Habener, 2009). Firstly, we quantified the total number of islets and the islet size distribution in WT and 6S-A mouse adult pancreata and we found no major differences (Figures S4A and S4B). We then measured the insulin- and glucagon-positive regions of the pancreas (as a percentage of total pancreas) and also found no significant differences (Figure S4C). We note that qPCR to detect hormone transcripts in isolated adult islets demonstrates a trend toward higher glucagon and Sst in the 6S-A Ngn3-expressing pancreata, yet neither reach statistical significance (p > 0.05) (Figure 3F). Thus, perturbation of the balance of endocrine subtypes brought about by preventing phosphorylation of Ngn3 can be at least partially compensated in the normal adult pancreas, which may reflect the previously described post-natal compensatory mechanisms that rebalance α and β cells (Collombat et al., 2009, Thorel et al., 2010).

### Ngn3 Phosphorylation Regulates Transcription of Endocrine Genes in Pancreatic Organoids

**Figure 4:**
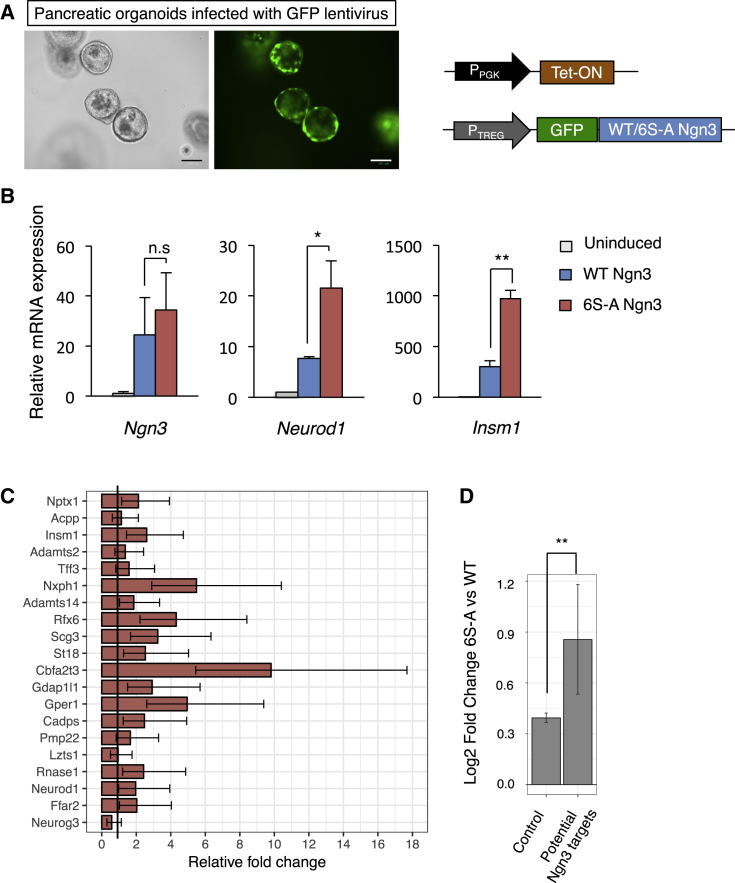
6S-A Ngn3 Shows Enhanced Target Gene Expression in Pancreatic Organoids (A) Inducible lentiviral vectors (illustrated) were used to infect ductal organoid cultures, images 2 days post-induction. Scale bars, 100 μm. (B) Relative mRNA expression of *Ngn3* and its downstream targets *Neurod1* and *Insm1* after 2 days of Ngn3 expression in organoids, normalized to β-actin. Data are mean ± SEM (n = 3). Student's t test, ^∗^p < 0.05, ^∗∗^p < 0.01. (C) Genome-wide transcriptomic analysis of pancreatic organoids expressing WT and 6S-A Ngn3. Graph showing the relative fold-change of expression in 6S-A Ngn3 organoids compared with WT Ngn3 fold-change (WT Ngn3 set as 1 unit) at 8 days after Ngn3 induction. Data represent mean fold change ± SEM (n = 3). (D) Average relative log2 fold-change of expression in potential Ngn3 targets compared with control (all genes excluding Ngn3 targets) at 8 days. Data represent average log2 fold change and error bars represent 95% confidence intervals of the mean (n = 3). ^∗∗^p < 0.01. See also Figure S5.

Cell proliferation, and the Cdk activity that drives it, is generally associated with maintenance of progenitors, while cell-cycle lengthening and exit with a corresponding reduction of Cdk activity, accompanies differentiation (Hardwick et al., 2015, Hardwick and Philpott, 2014). We see that 6S-A Ngn3 causes an increase in glucagon-expressing α cells when expressed from the endogenous locus (Figures 3B–3D). As α cells are the first endocrine cell type to be generated during pancreatic development, the increase in α cells would be consistent with an accelerated differentiation activity of 6S-A Ngn3. We therefore wanted to investigate whether preventing Cdk-dependent phosphorylation of Ngn3 might enhance expression of downstream targets of Ngn3 that potentiate the differentiation program. To this end, we tested the relative ability of WT and 6S-A Ngn3 to activate endocrine differentiation targets in 3D pancreatic ductal organoids, which exhibit characteristics typical of in vivo pancreatic progenitors such as extensive growth and endocrine differentiation potential (Huch et al., 2013). Induction of lentivirally transduced Ngn3 results in similar expression of WT Ngn3 and 6S-A Ngn3 mRNAs (Figures 4A and 4B). However, expression of *NeuroD1* and *Insm1*, downstream targets of Ngn3 that are key regulators of endocrine differentiation (Huang et al., 2000, Mellitzer et al., 2006), is 2- to 3-fold higher in 6S-A Ngn3-transduced organoids (Figure 4B).

To further characterize the Ngn3-driven transcriptional program that can be upregulated in ductal organoids, we sorted pools of 50 Ngn3-expressing cells and undertook genome-wide transcriptional profiling, determining gene expression at 2 and 8 days after upregulation of WT or 6S-A Ngn3. Ngn3 is a transcriptional activator, so we focused our analysis on the top 200 upregulated genes as potential Ngn3 targets. We found many genes that were previously described to play important roles in endocrine differentiation such as *Neurod1*, *Insm1*, *Atoh8*, and *Rfx6* (Figures 4C and S5; Table S1) (Smith et al., 2010). Activation of a pro-endocrine differentiation program is also confirmed by gene ontology analysis (Table S1), while other terms indicate upregulation of genes involved in neuronal differentiation, reflecting the close parallels between Neurogenin-driven differentiation in the two tissues (Otter and Lammert, 2016, van Arensbergen et al., 2010). Importantly, we see that 6S-A Ngn3 drives expression of the same genes as WT Ngn3, but it does so to a consistently higher level (p ≤ 0.005) (Figure 4D), an effect that is magnified between 2 and 8 days post-expression (Figures 4C, 4D, and S5). Thus, preventing Cdk-mediated phosphorylation of Ngn3 results in an enhancement of its ability to upregulate transcriptional targets that drive the endocrine differentiation program. We next investigated the mechanistic basis for the enhanced activity of un(der)phosphorylated Ngn3.

### Phosphorylation of Ngn3 Regulates Protein Stability and Binding to Targets

**Figure 5:**
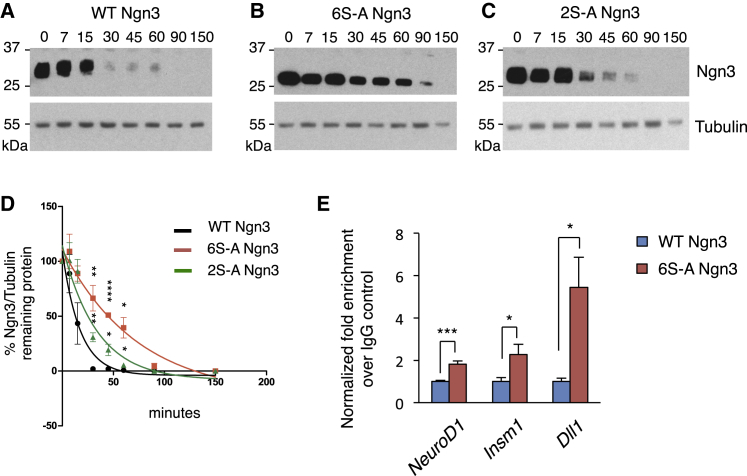
Ngn3 Phosphorylation Controls Protein Stability and Binding to Target Genes (A–C) HA-tagged WT Ngn3 (A), 6S-A (B), and 2S-A Ngn3 (C) protein expression following cycloheximide (CHX) addition (in min). (D) Graph showing degradation rate for different Ngn3 mutants, normalized to tubulin. n = 3 independent experiments, mean ± SEM. Student's t test, ^∗^p < 0.05, ^∗∗^p < 0.01, ^∗∗∗∗^p < 0.0001. (E) Chromatin immunoprecipitation (ChIP) from ductal mPAC cell extracts containing normalized amounts of HA-tagged Ngn3 WT and 6S-A. Data represent mean ± SEM. (n ≥ 3) Student's t test, ^∗^p < 0.05, ^∗∗∗^p < 0.001. See also Figure S6.

Enhanced activity of 6S-A Ngn3 protein could reflect increased stability (Sancho et al., 2014) or enhanced association with the regulatory regions of downstream target genes, or both. Phosphorylation-dependent recruitment of E3 ubiquitin ligases to bHLH transcription factors has been shown to control protein degradation (Forget et al., 2014, Sancho et al., 2014). In insulinoma-derived MIN6 cells, we see that 6S-A Ngn3 is five times as stable as WT Ngn3 (half-life 52.3 ± 7.9 min compared with 11.7 ± 2.8 min) (Figures 5A, 5B, and 5D). Phosphorylation of two serines of Ngn3 (mutated here in 2S-A Ngn3) promotes Ngn3 degradation via recruitment of the E3 ubiquitin ligase Fbxw7 (Sancho et al., 2014), and one of these sites is also mutated in 6S-A Ngn3 (Figure 1B). Comparing the stability of these proteins, 2S-A Ngn3 is twice as stable as WT Ngn3 (half-life of 23.1 ± 2.8 min compared with 11.7 ± 2.8 min), but considerably less stable than 6S-A Ngn3 (half-life 52.3 ± 7.9 min) (Figures 5C and 5D), showing that sites additional to those mutated in 2S-A Ngn3 contribute to the phosphorylation-dependent regulation of Ngn3 protein stability.

To investigate whether enhanced transcription of targets by 6S-A Ngn3 is solely due to increased half-life, we expressed WT or 6S-A Ngn3 in mPAC cells and determined Ngn3 binding at key target genes by chromatin immunoprecipitation (ChIP), after normalizing for the amount of chromatin-bound Ngn3 protein (Figure S6A). Even when normalized for protein level, 6S-A Ngn3 binding is more than 2-fold greater than WT Ngn3 on promoters of *Insm1* and *NeuroD1*, and 5-fold greater at predicted target E boxes of the Delta1 promoter (Figure 5E). Hence, enhanced protein stability and increased binding to downstream regulatory elements both contribute to the increased transcriptional activity of 6S-A Ngn3.

The enhanced *NeuroD1* and *Insm1* expression in response to 6S-A Ngn3 might explain the increased generation of α cells observed during embryonic development, which would be consistent with an early entry into the differentiation program (Figures 3B–3E). However, in addition to more early-born α cells, 6S-A Ngn3 mice also show enhanced Sst expression despite the fact that δ cells arise later in development (Figures 3B–3E). Ngn3 expression alone is sufficient to reprogram acinar cells into δ cells (Li et al., 2014). We determined by ChIP that Ngn3 binds directly to two consecutive E boxes found in the 3′ genomic region of *Sst* (Figure S6B), and this binding is increased 6.9- ± 2.3-fold by 6S-A Ngn3 compared with WT Ngn3 (Figure S6C). Moreover, we also see that *Sst* mRNA is upregulated by Ngn3 in this cell line (Figure S6D), indicating that *Sst* is a bone fide direct target of Ngn3. Therefore, the increased expression of *Sst* in the 6S-A Ngn3 E16 pancreas is likely to reflect enhanced *Sst* promoter binding and activation of 6S-A Ngn3 compared with WT Ngn3.

### Un(der)phosphorylated Ngn3 Maintains Adult β Cell Mature Identity in the Presence of Elevated c-Myc

**Figure 6:**
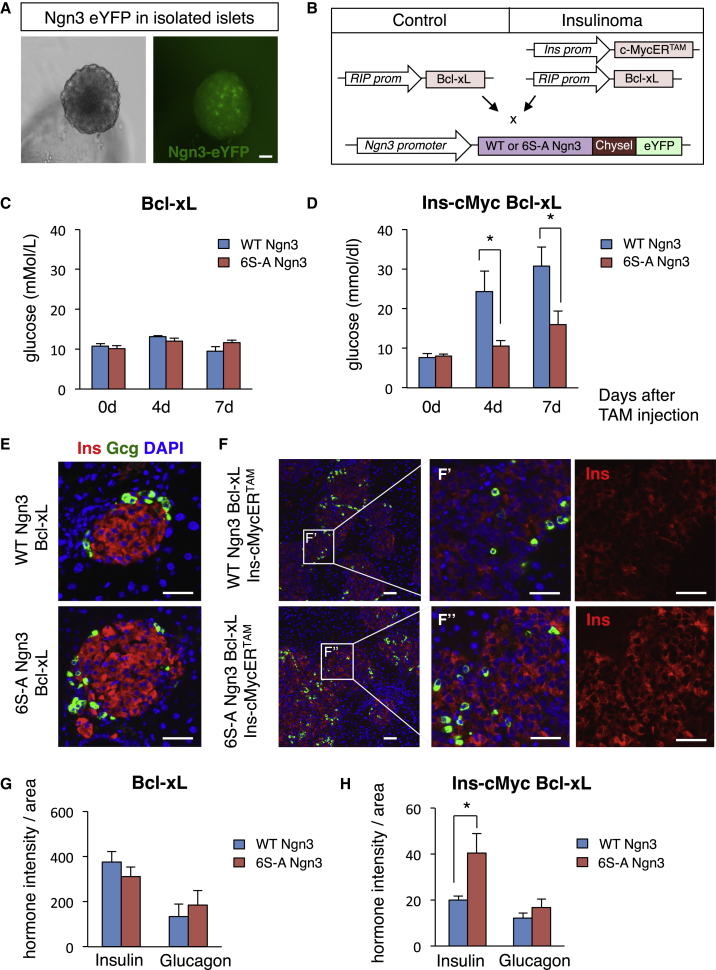
6S-A Ngn3 Maintains Insulin Expression in Adult β Cells Expressing c-Myc (A) eYFP expression in live culture of Ngn3-eYFP islets. Scale bar, 40 μm. (B) Schematic representation of animal models used for in vivo insulinoma analysis. (C and D) Quantification of blood glucose measurements (in mmol/L) in Bcl-xL (C) and Ins-cMycER^TAM^ Bcl-xL (D) mice at 0, 4, and 7 days of tamoxifen treatment. n ≥ 4 mean ± SEM. Student's t test, ^∗^p < 0.05. (E and F) Immunohistochemistry to detect insulin (red) and glucagon (green) in adult pancreatic sections from Bcl-xL only (E) or Ins-cMycER^TAM^ Bcl-xL (F) animals crossed with WT or 6S-A Ngn3 mice, as labeled, at 7 days of tamoxifen. Nuclei were counterstained with DAPI. Scale bar, 50 μm in (E, F, F′, and F’’). (G and H) Quantification of hormone intensity, divided by islet area in Bcl-xL (G) and Ins-cMycER^TAM^ Bcl-xL crossed with WT or 6S-A Ngn3 mice, as indicated (H) at 7 days of tamoxifen. Data represent mean ± SEM. n ≥ 3 different animals (islet analyses, 2–6 different sections for each animal for each condition, see STAR Methods for details). Student's t test, ^∗^p < 0.05. See also Figure S7.

Ngn3 is expressed at high levels during development and its expression is maintained at low levels in adult β cells (Figure 6A), where it is still required to maintain efficient function (Wang et al., 2009). Following on from our results demonstrating the enhanced capacity of 6S-A Ngn3 to promote differentiation in development and in organoid culture, we next investigated whether Ngn3 phosphorylation influences adult endocrine cell differentiation in islets that have been stimulated to proliferate. C-Myc upregulation in adult islets in a mouse model of insulinoma has a potent pro-proliferative effect and also results in loss of insulin expression and a failure to maintain blood glucose homeostasis (Pelengaris et al., 2002).

In the Ins-cMycER^TAM^ model that we have used (Pelengaris et al., 2002), rapid c-Myc activation occurs in islets in response to tamoxifen and under the control of the insulin promoter; extensive β cell apoptosis results, but this can be counteracted by co-expression of the apoptotic inhibitor Bcl-xL. To determine whether the phospho-status of Ngn3 could influence adult islet cell function, we compared Ins-cMycER^TAM^ Bcl-xL mice that were also homozygous for WT or 6S-A Ngn3, using Bcl-xL only mice as a control (Figure 6B).

In this model, over a 7 day period Ins-cMycER^TAM^ Bcl-xL islet expansion is accompanied by reduced insulin expression in β cells with accompanying hyperglycemia (Pelengaris et al., 2002) (Figure 6D). We compared glucose homeostasis 0, 4, and 7 days after tamoxifen-induction of c-Myc in WT Ngn3- and 6S-A Ngn3-expressing mice. In control conditions (BclX-xL WT or 6S-A Ngn3), glycemic values vary within the normal range, between 9.5 and 13.1 mmol/L (Figure 6C), while c-Myc induction in Ins-cMycER^TAM^ Bcl-xL WT Ngn3 raises blood glucose levels after 4 and 7 days of tamoxifen treatment to 24.25 ± 5.2 and 30.7 ± 4.9 mmol/L, respectively. Strikingly however, Ins-cMycER^TAM^ Bcl-xL 6S-A Ngn3 mice maintain glucose levels within the normal range (10.6 ± 1.3 mmol/L at 4 days and 16 ± 3.3 mmol/L at 7 days post-induction) (Figure 6D). Perhaps surprisingly, we see that massive islet expansion occurs to an equal extent in Bcl-xL WT Ngn3 and Bcl-xL 6S-A Ngn3 animals treated with tamoxifen for 7 days (Figures S7A–S7C). Thus, islet expansion occurs in response to c-Myc in Ins-cMycER^TAM^ Bcl-xL 6S-A Ngn3 mice, demonstrating that β cells expressing 6S-A Ngn3 can still proliferate. However, even though c-Myc can drive β cell expansion in both WT and 6S-A Ngn3 mice, blood glucose control is only maintained in 6S-A Ngn3 mice. Therefore, blocking its phosphorylation allows Ngn3 to maintain differentiated function of β cells even while they are actively proliferating.

To investigate directly whether the expanded islets in 6S-A Ngn3 mice could maintain insulin expression even in the face of c-Myc overexpression, we used immunohistochemistry to detect insulin protein. After quantitation of hormone intensity in stained islets (see STAR Methods for details of analysis), we find that insulin and glucagon levels are very similar in the control Bcl-xL WT Ngn3 and Bcl-xL 6S-A Ngn3 animals after 7 days of tamoxifen (Figures 6E–6G). However, Ins-cMycER^TAM^ Bcl-xL 6S-A Ngn3 animals maintain insulin expression at twice the level of Ins-cMycERT Bcl-xL WT Ngn3 mice (a relative intensity of 40.5 ± 8.4 in Ins-cMycERT; Bcl-xL; 6S-A Ngn3 compared with 20 ± 1.7 in Ins-cMycERT; Bcl-xL; WT Ngn3) (Figures 6F–6H). No differences in glucagon expression are observed, consistent with the fact that Ngn3 is not known to be expressed in α cells. Thus, results from 6S-A Ngn3 mice suggest enhanced expression of insulin in proliferating islets compared with WT mice, while showing no differences in expression of glucagon, indicating a cell-autonomous effect. However, further experiments looking directly at insulin secretion would be needed to confirm that enhanced insulin production arising from dephosphorylation of Ngn3 is responsible for maintenance of glucose homeostasis in Ins-cMycERT; Bcl-xL; 6S-A Ngn3 mice.

To investigate more directly whether preventing Ngn3 phosphorylation limits c-Myc-mediated inhibition of Ngn3 activity in another context, we tested the ability of WT and 6S-A Ngn3 to activate *Insm1* and *Neurod1* in *Xenopus* embryos, with and without co-injected c-Myc. As we saw in mammalian cells, ectopic 6S-A Ngn3 resulted in greater expression of *Insm1* and *Neurod1* than WT Ngn3. C-Myc overexpression suppresses Ngn3-driven *Neurod1* and *Insm1* expression, but levels nevertheless remain higher with 6S-A Ngn3 compared with WT Ngn3 (Figure S7D). As Ngn3 and c-Myc are co-injected, this is likely to represent a cell-autonomous effect.

### Regulation of Cell-Cycle Exit by Ngn3 Phosphorylation

**Figure 7:**
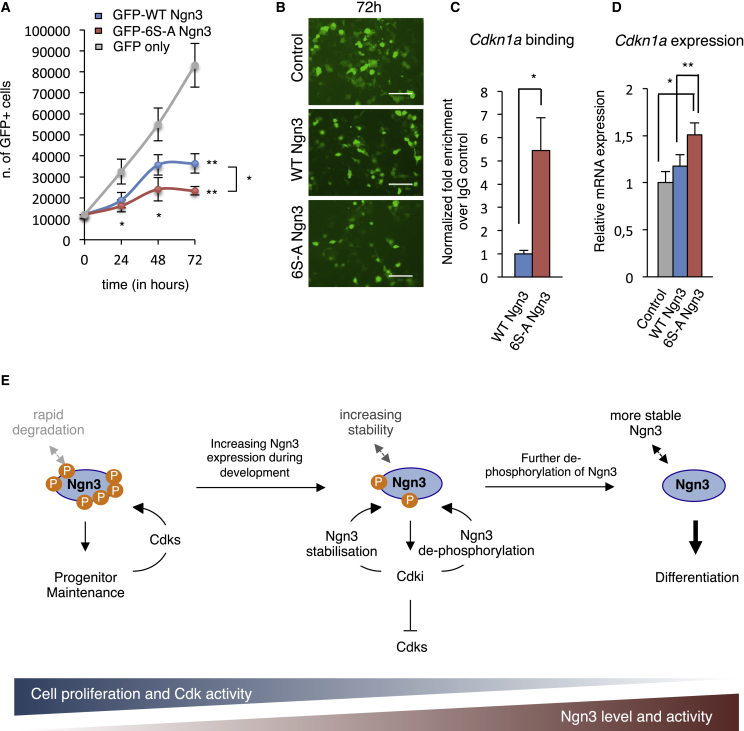
Ngn3 Dephosphorylation Enhances Cell-Cycle Exit in Pancreatic Ductal Cells (A) Graph showing the growth of pancreatic ductal mPAC cells at 24, 48, and 72 hr after transfection with WT and 6S-A Ngn3 and GFP or GFP only, counting GFP+ cells. Data are mean ± SEM of four independent experiments. Student's t test, ^∗^p < 0.05, ^∗∗^p < 0.01. (B) Representative images of mPAC cells 72 hr after transfection. Scale bar, 100 μm. (C) Chromatin immunoprecipitation (ChIP) from ductal mPAC cell extracts expressing HA-tagged WT and 6S-A Ngn3. Data represent mean ± SEM (n ≥ 3). Student's t test, ^∗^p < 0.05. (D) Relative mRNA expression of *Cdkn1a* after 2 days of Ngn3 expression in ductal PAC cells, normalized to β-actin. Data are mean ± SEM (n = 3). Student's t test, ^∗^p < 0.05, ^∗∗^p < 0.01. (E) Model illustrating how Ngn3 phosphorylation controls the balance between proliferation and differentiation during development of the endocrine pancreas. Cdk, cyclin-dependent kinase; Cdki, Cdk inhibitor; P, phosphorylation.

A recent study demonstrates that a low level of Ngn3 in pancreatic endocrine cell progenitors is compatible with continued proliferation and maintenance of a precursor state (Bechard et al., 2016). However, increasing Ngn3 expression drives endocrine commitment, cell-cycle exit, and finally endocrine cell differentiation (Bechard et al., 2016, Miyatsuka et al., 2011). As well as potentiating endocrine differentiation, we see that 6S-A Ngn3 drives cell-cycle exit more effectively than WT Ngn3 in mPAC cells (Figure 7A and 7B). Since Ngn3 is known to drive the cell cycle in endocrine progenitors by upregulating Cdkn1a expression (Miyatsuka et al., 2011), an inhibitor primarily of Cdk2, we investigated Cdkn1a regulation by WT and 6S-A Ngn3. 6S-A Ngn3 binds to the Cdkn1a distal regulatory region (Miyatsuka et al., 2011) (Figure 7C), and activates Cdkn1a expression to a greater extent than WT Ngn3 (Figure 7D), consistent with its enhanced ability to drive cell-cycle exit (Figures 7A–7D). Hence, Ngn3 both drives Cdk inhibition and cell-cycle exit as well as responding to it, because of an enhanced ability of un(der)phosphorylated Ngn3 to upregulate the endocrine differentiation program (Figure 7E).

## Discussion

The role of *Ngn3* in pancreatic endocrine cell fate specification and differentiation has been extensively studied, but surprisingly little is known about the activity and regulation of Ngn3 protein (Roark et al., 2012, Rukstalis and Habener, 2009, Sancho et al., 2014). Here we show that Ngn3 is phosphorylated on up to six sites by Cdks. Inhibition of Cdk-dependent phosphorylation enhances pancreatic endocrine differentiation both by stabilizing Ngn3 protein and additionally by enhancing its ability to bind to downstream targets. Importantly, we show that preventing phosphorylation of Ngn3 expressed at the endogenous level is sufficient to promote enhanced differentiation of glucagon- and somatostatin-expressing cells in the developing pancreas. Furthermore, even in the face of forced c-Myc expression and a significant drive toward decreased insulin production, 6S-A Ngn3 expressed from the endogenous promoter is sufficient to maintain β cell function.

Our data allow us to propose a model of Ngn3 post-translational regulation that can explain how endocrine precursors transition from proliferating progenitors to endocrine cells firmly committed to undergoing differentiation (Figure 7E). In proliferating progenitors, Ngn3 expression levels are low and Cdk levels are high, resulting in extensive Ngn3 phosphorylation. Low levels of phosphorylated Ngn3 are compatible with, and may be required for, endocrine progenitor maintenance (Bechard et al., 2016). Firm endocrine commitment coincides with a rise in Ngn3 transcripts (Bechard et al., 2016), and this rising Ngn3 results in increased expression of its direct target Cdkn1a (Figures 7C and 7D), which predominantly inhibits Cdk2. Cdk inhibition then results in both slowing of the cell cycle and in limiting the Cdk-dependent phosphorylation of Ngn3. Moreover, Ngn3 is directly stabilized by the Cdkn1a homolog p27Xic1 in *Xenopus* (Roark et al., 2012). Therefore, as cells pass through endocrine commitment with increasing Cdkn1a levels and falling Cdk activity, Ngn3 protein levels will rise through a combination of elevated expression (Bechard et al., 2016) and enhanced protein stability, while its association with regulatory regions of downstream targets driving differentiation will also increase. This rise in un(der)phosphorylated Ngn3 activity drives further increases in activation of downstream targets of Ngn3 such as *Cdkn1a*, *Insm1*, and *Neurod1*, ultimately leading to cell-cycle exit, endocrine commitment, and then differentiation in a feedforward loop.

Ngn3 expression is highest at endocrine commitment in development, with levels dropping dramatically after this point coinciding with terminal differentiation (Villasenor et al., 2008, Zhou et al., 2008). Our model of Ngn3 phosphoregulation coupling cell cycle and differentiation can help to explain the transition from proliferating progenitor to differentiating endocrine cell during development when Ngn3 is highly expressed. However, whether the same coupling mechanisms are maintained in adults in homeostasis or during tissue repair has been unclear.

Ngn3 is required for adult β cell function (Wang et al., 2009) and we see that phosphorylation of Ngn3 may play a regulatory role in maintaining β cell function in the presence of pathological pro-proliferative cues. In a model for the human islet cancer insulinoma, β cells can be driven to proliferate by c-Myc overexpression and this is usually accompanied by loss of insulin expression (Pelengaris et al., 2002). However, 6S-A Ngn3-expressing islets maintain insulin expression and support glucose homeostasis while still undergoing massive islet expansion (Figures 6 and S7). Interestingly, this demonstrates that expression of un(der)phosphorylated Ngn3 alone is not sufficient to counteract c-Myc-driven cell division and that even a high rate of β cell proliferation is not fundamentally incompatible with differentiated function (Dor et al., 2004).

Conditional ablation of Ngn3 in adult β cells results in mild glucose intolerance and decreased expression of classical β cell markers, thus pointing toward an essential role for Ngn3 in maintaining mature β cell identity (Wang et al., 2009). There is also increasing evidence that β cell de-differentiation and a concomitant increase in Ngn3 expression may significantly contribute to the pathogenesis of diabetes (Talchai et al., 2012). Moreover, a very recent study has showed that subjecting diabetic mice to a fasting/refeeding regime results in regeneration of new β cells, a phenomenon that can reverse the diabetic phenotype (Cheng et al., 2017). In this model, more β cells arise after refeeding via a proliferating population of cells newly expressing elevated Ngn3, which in many ways resemble Ngn3-expressing developmental endocrine precursors (Bechard et al., 2016). These cells subsequently differentiate into insulin-secreting mature β cells. We would predict that Ngn3 dephosphorylation plays a role in transitioning from a precursor-like state to a functional β cell on refeeding. This study also shows that applying a “fasting-mimicking” growth factor medium to human type 1 diabetic islets induces expression of Ngn3 and insulin, but when insulin growth factor 1 (IGF-1) was added, Ngn3 expression remained but insulin was lost, essentially indicating a block of islet differentiation. It would be very interesting to determine whether Ngn3 is phosphorylated in these IGF-1-treated type 1 diabetic islets; IGF-1 has been shown to enhance mouse β cell proliferation (Agudo et al., 2008), which potentiates Ngn3 phosphorylation by Cdks, thus inhibiting its ability to drive and maintain β cell differentiation. Moreover, as many β cell-generating protocols transit a Ngn3-positive phase (Melton, 2016), it will be interesting to see whether dephosphorylation of endogenously expressed Ngn3 protein will similarly enhance differentiation and maturation of β cells generated in vitro.

bHLH transcription factors play a central role in progenitor maintenance and differentiation in the nervous system, pancreas, gut, and many other tissues. In most cases, the levels of bHLH gene expression have been well documented (Bertrand et al., 2002), but protein level and activity is much less clear. The multi-site phosphoregulation of Ngn3 we describe here is highly reminiscent of that seen in other proneural proteins that are master regulators of neurogenesis in the central and peripheral nervous systems. For instance, ectopic overexpression of a nine SP site phosphomutant form of Ngn2 in *Xenopus* results in substantially enhanced reprogramming of the epidermis into neurons compared with the WT protein (Ali et al., 2011), while Ngn2 protein is stabilized by the Cdkn1a homolog p27Xic1 (Nguyen et al., 2006, Vernon et al., 2003). Similar multi-site phosphorylation is also seen to inhibit the activity of ectopically expressed Ascl1 and NeuroD4 proteins (Ali et al., 2014, Hardwick and Philpott, 2015, Wylie et al., 2015). It seems likely that post-translational regulation by Cdks is a common mechanism at work widely among members of the bHLH family (Philpott, 2015). In these cases, and in the case of Ngn3 (Figures 4 and 5), an enhanced ability of un(der)phosphorylated proneural proteins to bind to the regulatory elements of their downstream targets may underlie much of their increased transcriptional activity, over and above any additional protein-stabilizing effect of dephosphorylation in vivo (Ali et al., 2011, Ali et al., 2014, Hardwick and Philpott, 2015, Hindley et al., 2012; our unpublished data). Overall, Ngn3 regulation may serve as a paradigm for ensuring the coordination of fate commitment, cell-cycle exit, and differentiation by bHLH transcription factors in response to the cellular environment in multiple tissues.

## STAR★Methods

### Key Resources Table

**Table:** 

REAGENT or RESOURCE	SOURCE	IDENTIFIER
**Antibodies**		

Guinea pig anti-insulin	Abcam	ab7842; RRID: AB_306130
Mouse anti-glucagon	Abcam	ab10988; RRID: AB_297642
Rabbit anti-somatostatin	Dako	A0566; RRID: AB_10013726
Goat anti-PPY	Abcam	ab77192; RRID: AB_1524152
Goat anti-Ngn3	Santa Cruz Biotechnology	sc-13793; RRID: AB_650136
Mouse anti-Ngn3	DSHB	F25A1B3; RRID: AB_528401
Rabbit anti-Ngn3	Gift of Dr. Edlund (Selander and Edlund, 2002)	N/A
Rat anti-HA HRP conjugated	Roche	12013819001; RRID: AB_390917
Rabbit anti-HA	Abcam	ab9110 RRID: AB_307019
Chicken anti-GFP	Abcam	ab13970 RRID: AB_300798
Mouse anti-Epcam APC	eBioscience	17-5791-80; RRID: AB_1659714

**Chemicals, Peptides, and Recombinant Proteins**		

Tamoxifen	Sigma-Aldrich	T5648
Lambda Protein Phosphatase	New England Biolabs	P0753S
Palbociclib isethionate	selleckchem	PD0332991
Roscovitine	selleckchem	S1153
CDK2/ CYCLIN A	Thermo Fisher Scientific	PV3267
CDK1/ CYCLIN B	Thermo Fisher Scientific	PV3292
CDK4/ CYCLIN D1	Thermo Fisher Scientific	PV4400
CDK2/ CYCLIN E1	Abcam	ab85639
EGF (organoid growth)	Invitrogen	PMG8043
FGF10 (organoid growth)	Peprotech	100-26
Gastrin (organoid growth)	Sigma	G9145
Noggin (organoid growth)	R&D System	1967
R-Spondin (organoid growth)	In house (Huch et al., 2013)	N/A

**Critical Commercial Assays**		

Rnaesy Mini Kit	Qiagen	74104
High-Capacity cDNA RT Kit	Thermo Fisher Scientific	4368814
QuantiTect Reverse Transcription kit	Qiagen	205310
LenitiX concentrator	Clontech	631231
LentiX Titration Kit	Clontech	631235
Advanced DMEM (organoid growth)	Gibco	12634
Low Glucose DMEM (islet isolation)	Media Tech	10-014-CM
Nextera Kit	Illumina	FC-131-1096
Illumina index	Illumina	FC-131-1002
Superscript II	Life Technolgies	18064-014

**Deposited Data**		

Organoid RNAseq data	GEO	GSE96707

**Experimental Models: Cell Lines**		

mPAC L20	Douglas Winton laboratory (Yoshida and Hanahan, 1994)	N/A
MIN6	Douglas Melton laboratory (Ishihara et al., 1993)	N/A

**Experimental Models: Organisms/Strains**		

Mouse InsMycER^TAM^: Tg(Ins-MYC/Er)1Gev	Gerard Evan laboratory	RRID: MGI: 3821935
Mouse: Tg(Ins1-BCL2L1)1Ksp	Gerard Evan laboratory	MGI: 2384544
Mouse: WT-Ngn3^eYFP^	Generated in our laboratory	N/A
Mouse: 6S-A Ngn3^eYFP^	Generated in our laboratory	N/A

**Recombinant DNA**		

pLVX-CMV-Tet3G	Clontech	631358
pLVX-TREG	Clontech	631193
pLVX-PGK-Tet3G	This paper	N/A
pCS2-Ngn3-HA	This paper	N/A
pCS2-6S-A Ngn3-HA	This paper	N/A
pCS2-2S-A Ngn3-HA	This paper	N/A
pLVX-TREG-GFP-P2A-WT Ngn3-HA	This paper	N/A
pLVX-TREG-GFP-P2A-6S-A Ngn3-HA	This paper	N/A

**Sequence-Based Reagents**		

Primers for gene expression, see Table S2	This paper	N/A
Taqman probes for gene expression, see Table S2	This paper	N/A
Primers for Chip, see Table S2	This paper	N/A
Primers for Xenopus experiment, see Table S2	This paper	N/A

**Software and Algorithms**		

ZEN Imaging software	Carl Zeiss	https://www.zeiss.com
Scran	Bioconductor	http://bioconductor.org/packages/release/bioc/html/scran.html
DESeq2	Bioconductor	https://bioconductor.org/packages/release/bioc/html/DESeq2.html

### Contact for Reagent and Resource Sharing

Further information and requests for reagents may be directed to, and will be fulfilled by, the Lead Contact, Anna Philpott (ap113@cam.ac.uk).

### Experimental Model and Subject Details

#### Mice

Mice were housed, bred and treated according to the Home Office guidelines, under the Animal Scientific Procedure Act (ASPA) 1986. All animal experiments were approved by the Animal Welfare and Ethical Review Body (AWERB) at the University of Cambridge. InsMycER^TAM^; Bcl-xL animals have been previously described (Pelengaris et al., 2002). Generation of WT Ngn3^eYFP^ and 6S-A Ngn3^eYFP^ mice is described in Figure S2. Knock-in WT Ngn3^eYFP^ and 6S-A Ngn3^eYFP^ animals were generated by embryonic stem cell targeting of the endogenous Ngn3 locus. A PGK-driven puromycine resistance cassette has been used to select targeted ES clones and removed after initial crossing with Flpase mice (Farley et al., 2000) The newly generated WT Ngn3^eYFP^ and 6S-A Ngn3^eYFP^ alleles have been constitutively recombined into the endogenous Ngn3 locus by crossing with a PGK-Cre line (Lallemand et al., 1998).

#### Xenopus laevis

*Xenopus laevis* were housed, bred and treated according to the Home Office guidelines, under the Animal Scientific Procedure Act (ASPA) 1986. All animal experiments were approved by the Animal Welfare and Ethical Review Body (AWERB) at the University of Cambridge. *Xenopus laevis* eggs and early stage embryos were obtained by standard methods (Vernon et al., 2003, Vosper et al., 2009).

#### Cell Lines

Immortalized cell lines (HEK293, HEK293T, mPAC L20, MIN6) were grown in Dulbecco’s modified eagle medium (DMEM) supplemented with Glutamax, 10% foetal bovine serum (FBS) and penicillin/streptomycin (Pen/Strep)

#### Primary Cell Cultures

Primary islet cultures were grown in low glucose (1g/L) DMEM supplemented with FBS and Pen/Strep. Primary ductal organoid cultures were seeded in matrigel and grown in Advanced DMEM media supplemented with N2 and B27 (Lifesciences), 1.25 mM N-Acetylcysteine (Sigma), 10 nM gastrin (Sigma) and the following growth factors: 50 ng/ml EGF (Peprotech), 5% RSPO1-conditioned media, 25 ng/ml Noggin (R&D System), 100 ng/ml FGF10 (Peprotech) and 10 mM Nicotinamide (Sigma) (Huch et al., 2013).

### Method Details

#### Mouse Genotyping

Recombinant eYFP-containing alleles were amplified using the following primers: forward, Ngn3-Fw 5’-TACATCTGGGCACTGACTCAGA-3’ and reverse Ngn3-eYFP-Rv 5’-GTCGTCCTTGAAGAAGATGGTG-3’; un-recombined wild-type allele was identified using the same forward primer and the reverse primer Ngn3-Rv 5’-CTTGGAGCGAGAGTTTGATGTG-3’. PCR condition were 30 cycles of 94^o^C/10sec; 60^o^C/20sec; 72^o^C/30sec. Distinction between WT and 6SA Ngn3 has been performed by sequencing the amplified PCR product with the primer 5’-CATAGCGGACCACAGCTTCT-3’ or by cutting with restriction enzyme BglI.

#### Plasmid Constructs

Phosphomutant versions of Ngn3 were obtained by site-directed mutagenesis (QuickChange II Site-Directed Mutagenesis Kit, Stratagene) of a murine pCS2-Ngn3 expression plasmid. 6S-A Ngn3 carries mutations in positions S14, S38, S160, S174, S183 and S199. 2S-A Ngn3 exhibits mutations in S183 and S187. HA-tagged wild type and mutants Ngn3 have been cloned by introducing the following sequence at Ngn3 C-terminus: 5’-TACCCATACGATGTTCCAGATTACGCTTAA-3’. Lentiviral vectors have been obtained by subcloning Ngn3 coding sequence into pLVX-TREG (Clontech).

#### Protein Phosphorylation Analysis

In vitro translated (IVT) radiolabelled proteins were incubated with interphase (I) or mitotic (M) *Xenopus* egg extracts. HA-tagged WT and 6S-A Ngn3 proteins were collected 24 hours post transfection and analysed by SDS-PAGE, using rat anti-HA-Peroxidase (1:1000; Roche) and mouse anti-tubulin (1:1000, Sigma). CyclinB Δ90 was produce in E. Coli, purified and incubated with IVT Ngn3 at 21°C for 40 minutes. Endogenous Ngn3 from E14 mouse pancreas was detected with rabbit anti-Ngn3 antibody (1:600; kind gift from Dr Helena Edlund, Figures 1F and S1C) or mouse anti-Ngn3 (1:500, Developmental Studies Hybridoma Bank, Figure S2B). Phosphatase treatment was performed by 30-minute incubation at 30°C with 400 units of Lambda Protein Phosphatase (NEB). Pancreatic ductal mPAC cells expressing HA-tagged WT-Ngn3 were treated with Roscovitine (Selleckchem) and Palbociclib (Selleckchem) at different concentrations for 24 hours.

#### Protein Stability Assay

Mouse insulinoma MIN6 cells overexpressing WT, 2S-A and 6S-A Ngn3 were treated with cycloheximide (10μg/ml). Proteins were collected at different time points after treatment and lysed in RIPA-like lysis buffer (50mM Tris-HCl, pH 8; 150mM NaCl; 0.5% NP40 Igepal; 10% Glycerol; protease and phosphatase inhibitor cocktails (Roche; Calbiochem)). Proteins were separated on SDS-PAGE and immmunoblotted with rat anti-HA-Peroxidase (1:1000; Roche) and mouse anti-tubulin (1:1000, Sigma). Protein levels were quantified with ImageJ software (n=3). Half-lives were calculated using first-order kinetics (Prism).

#### *In Vitro* Kinase Assay

In vitro kinase assay has been performed by incubating in vitro translated HA-tagged WT and 6S-A Ngn3 proteins with human recombinant CDK/CYCLINs (0.5μM final concentration of CDK4/CYCLIND1, CDK2/CYCLINA, CDK1/CYCLINB from ThermoFisher; CDK2/CYCLINE1 from Abcam) in the presence of 10 μM ATP for 1 hour at 30°C. In vitro translation was performed in the presence of 800mM LiCl to reduce potential phosphorylation in reticulocyte lysate. Samples were resolved by SDS-PAGE and protein detected with anti-HA antibody.

#### Pancreatic Organoid Cell Culture

Pancreatic ducts were isolated from the pancreas of adult mice. Pancreatic digestion, duct isolation and organoid culture were performed as previously described (Huch et al., 2013). Briefly, chopped pancreas was incubated for 45-60 minutes at 37° C in collagenase/dispase dissociation Medium (1% in DMEM media (Gibco), supplemented with 1% FBS (Gibco) and Collagenase type XI 0.012% (w/v) (Sigma), dispase 0.012% (w/v) (Gibco)). Single ducts were manually picked, re-suspended with Matrigel (BD Bioscience) and seeded. Culture media is composed of AdDMEM/F12 (Invitrogen) supplemented with N2 and B27 (Lifesciences), 1.25mM N-Acetylcysteine (Sigma), 10nM gastrin (Sigma) and the following growth factors: 50 ng/ml EGF (Peprotech), 5% RSPO1-conditioned media, 25ng/ml Noggin (R&D System), 100 ng/ml FGF10 (Peprotech) and 10mM Nicotinamide (Sigma).

#### Infection of Pancreatic Organoids

Pancreatic organoids were infected with a two-vector lentivirus-based Tet-on system (Clontech). Coding sequences for HA-tagged WT and 6S-A Ngn3, fused with GFP-2A cleavage peptide sequence at their N-terminus, were cloned into the pLVX-TRE3G vector (Clontech). Viruses were generated in HEK293T cells, titrated with the LentiX titration kit (Clontech) and used at multiplicity of infection of 10 for the transgene, or 20 for the transctivator Tet3G. Organoids were dissociated to small clusters by TrypLE (Gibco) treatment for 10 min at 37^0^C. Dissociated organoids were incubated with the viruses and 8μg/ml polybrene (Sigma) in expansion media supplemented with 10μM ROCKI (Sigma) and spun for 1h at 300xG at room temperature. After spinoculation, infected organoids were incubated in a cell culture incubator at 37^0^C for 5-6 hours before plating in matrigel with fresh media supplemented with ROCKi.

#### RNA Sequencing of Pancreatic Organoids

Pancreatic organoids infected with GFP-Ngn3 (WT/6S-A) were dissociated to single cells by TrypLE treatment for 10 min at 37^0^C. Pools of 50 GFP+ cells were FACS sorted and collected into a 96 well plate containing lysis buffer. Library preparation for RNA sequencing used the protocol for single cell RNA sequencing described and optimized in (Picelli et al., 2014, Wilson et al., 2015). The Illumina Nextera XT DNA preparation kit was used to prepare libraries. Pooled libraries were sequenced using the Illumina HiSeq 4000 system (single-end 50 bp reads).

#### Islet Isolation

Mouse pancreatic islet isolation was performed as previously described (Li et al., 2009) with some modifications. Mice were euthanized in accordance with ASPA 1986 guidelines. The abdominal internal region was exposed and in situ pancreatic digestion was performed: a clamp was positioned on the top of the duodenal papilla to block the passage from the common bile and pancreatic duct into the intestine. Pancreatic digestion was carried out by injecting collagenase-containing media (Low glucose DMEM (Mediatech), 10mM Hepes buffer (Invitrogen), collagenase 0.8 mg/ml (Roche)) into the ductal trees. Inflated pancreata were then collected and incubated at 37^o^C for 15 minutes in the collagenase media. Islet purification was performed by sequential filtration through a 250μm filter and density gradient cell separation using a combination of Histopaque media (H-1077 and H-1119, Sigma). Single acinar cells and cell clusters smaller than 40μm were removed and pancreatic islets were manually handpicked. After O/N 37^o^C incubation in culture media, islets were lysed for RNA extraction.

#### Immunohistochemistry

Embryonic stage 16 pancreatic buds were fixed in 4% paraformaldehyde (PFA) for 10 minutes at 4C, embedded in OCT and sectioned at 10μm thickness using a cryostat (Leica). Primary antibodies: Insulin (guinea pig, 1:200, Abcam), Glucagon (mouse, 1:200, Abcam), Somatostatin (rabbit, 1:200, Dako), Ppy (goat, 1:200, Abcam), GFP (chicken, 1:600, Abcam), Ngn3 (goat, 1:100 Santa Cruz), Epcam (APC conjugated, 1:600, eBioscience). Adult pancreata were dissected from age-matched animals older than 6 weeks and fixed in 4% formalin (Sigma) O/N 4C. Pancreata were paraffin embedded and sectioned at 5μm thickness, 100μm apart.

#### Image Acquisition

Bright field and eGFP pictures of live cultures were taken with EVOS (LifeTechnologies) and ZOE (Biorad). Immunofluorescence pictures of embryonic buds and adult pancreata were imaged using an automated system (Axioscan, Zeiss).

#### Analysis of Insulinoma Mouse Model

Adult Ins-cMycER^TAM^; Bcl-xL and the control Bcl-xL mice were injected IP with 1mg of Tamoxifen (Sigma) in oil every day for 7 days. Blood glucose was measured after a nick in the tail vein using AlphaTRAK (Abbott). Pancreata were analysed as described in Quantification and Statistical analysis.

#### *Xenopus laevis* Gene Expression

*Xenopus laevis* eggs were obtained by standard hormone methods of induction, and subsequently fertilised in vitro. WT, 6S-A Ngn3 and c-Myc mRNAs were transcribed in vitro using the SP6 mMessage mMachine® kit (Ambion). Two-cell-stage embryos were injected into the animal pole with mRNA as indicated (0.75pg WT/6S-A Ngn3, 750pg c-Myc), with co-injection of 500 pg GFP to confirm successful targeting. Embryos were subsequently cultured at 16°C in Ficoll solution (4 % w/v Ficoll, 0.2x MBS, 50μg/ml Gentamycin in water) and staged according to the methods of Niewkoop and Faber. At stage 18, samples of four embryos were snap-frozen for qRT-PCR analysis. Primer list is provided in Table S2.

#### Real Time PCR

Gene expression in organoid cultures, isolated islets and *Xenopus laevis* embryos were performed by real-time semi-quantitative PCR. RNA was extracted with RNAesy mini kit (Qiagen) and retro-transcribed using QuantiTect Reverse Transcription kit (Qiagen; for islets and *Xenopus* embryos) or High-Capacity cDNA Reverse Transcription Kit (Applied Biosystem; for organoids). Real time PCR was performed with QuantiFast SYBR Green PCR kit (Qiagen; for islets and *Xenopus* embryos) or with Taqman probes (Applied Biosystem; for organoids). LightCycler (Roche) was used to run the PCR and analyse the data. EF1α or ActinB were used for endogenous reference gene control and the values were normalized to control levels. Relative quantification was determined according to the DDc(t) method. Data are presented as means ± s.e.m. of normalized values from three independent experiments, unless otherwise stated. The list of primers and Taqman probes is provided in Table S2.

#### Chromatin Immunoprecipitation (ChIP)

ChIP experiments were performed on mPAC cell extracts, 24 hours after transfection with either HA-tagged WT or 6S-A Ngn3. Chromatin-protein complexes were cross-linked with 1% formaldehyde. Five micrograms of rabbit anti-HA antibody (Abcam) or control anti-IgG (Abcam) were used per ChIP reaction and quantified using SYBR Green mix. The signal over background normalisation method was used to quantify immunoprecipitated DNA. Primer list is provided in Table S2.

### Quantification and Statistical Analysis

Figure Legends describe the statistical test and the associated parameters used to analyse the data. Asterisks indicate statistical significance as explained in each figure legend (^∗^, p < 0.05; ^∗∗^, p < 0.01; ^∗∗∗^, p < 0.001; ^∗∗∗∗^, p < 0.0001).

#### Analysis of Embryonic and Adult Pancreas

The percentage of hormone positive areas in the embryonic pancreatic buds was quantified using ZEN software (Zeiss). Values are shown as means ± s.e.m of 4 pancreatic buds from 3 different litters. Insulin and glucagon positive areas in the adult pancreas were calculated using ZEN software (Zeiss) and shown as a percentage over total DAPI area. Values are means ± s.e.m n=4 from 6-8 sections for each animal, 100μm apart. Total number of islets and islet size distribution were calculated using ZEN software (Zeiss). The optimized ZEN programme calculated islet size by creating a mask over insulin plus glucagon merged areas. Values are means ± s.e.m n=4 from 6-8 sections for each animal, 100μm apart.

#### Analysis of the Insulinoma Pancreata

Hormone intensity in the Ins-cMycER^TAM^; Bcl-xL and Bcl-xL animals was calculated using ZEN software (Zeiss) and normalized for the total islet area analyzed. At least 100 islets per condition were quantified, coming from 2-6 different sections (100μm apart) for each animal from at least 3 different animals. Values are means ± s.e.m n≥3 different animals for each genotype. Islet area was calculated using ZEN software (Zeiss) from H&E stained tissue sections. n≥3 different animals each genotype (2-11 sections each animal).

#### RNA Sequencing Analysis

Reads were aligned using G-SNAP 30 and the mapped reads were assigned to Ensembl genes (release 81) by HTSeq. To identify poor quality samples, three metrics were used: (1) the proportion of reads aligned to spike ins, (2) the number of endogenous reads, and (3) the number of features with more than 0 read. We filtered for cells with (1) less than 30% reads aligned to spike ins, (2) more than 1000000 endogenous reads, and (3) more than 10000 detected features. We only considered genes that were detected in at least 5 samples (including technical and biological repeats), with a variance greater than 0. Based on this, two samples were not considered for downstream analysis. Reads were normalised using size factor normalisations as implemented in the scran package (Lun et al., 2016) (*scran: Methods for Single-Cell RNA-Seq Data Analysis*. R package version 1.2.0). Differential expression analysis was performed using the DESeq2 package, where technical repeats were merged (Love et al., 2014).

### Data and Software Availability

#### Deposited Data

The accession number for the sequencing data reported in this paper is GEO: GSE96707.

## Author Contributions

A.P. conceived the initial study. R.A. and A.P. designed the study with contributions from D.W., B.D.S., G.E., M.H., and B.G. R.A. and A.P. wrote the manuscript. R.A. performed phosphorylation and stability assays, Cdk inhibitor treatment, in vitro kinase assay, and adult pancreas, organoid, insulinoma, and cell-cycle exit experiments. C. Hurley, D.W., R.K., and K.J. developed the 6S-A Ngn3 mouse model. I.G. and F.M. contributed to Figures 6C and 6D. M.K.S. undertook embryonic analysis. L.M. contributed to Figure S3. F.A. performed ChIP. C. Hurley and L.H. performed *Xenopus* experiments. C. Hindley and M.H. contributed to organoid derivation. S.R. performed bioinformatic analysis. S.N contributed to library preparation for sequencing. All authors discussed the results and commented on the manuscript.

## Supplemental Information

Document S1. Figures S1–S7

Table S1. Genome-wide Transcriptomic Analysis of Pancreatic Organoids Expressing WT and 6S-A Ngn3

Table S2. List of Primers

Document S2. Article plus Supplemental Information
