# Supplementary material for: Multi-site Neurogenin3 Phosphorylation Controls Pancreatic Endocrine Differentiation
Source: Dev Cell. 2017 May 8;41(3):274–286.e5. doi: 10.1016/j.devcel.2017.04.004 (PMC5425251; doi:10.1016/j.devcel.2017.04.004)
Supplement: Document S1. Figures S1–S7 [file mmc1.pdf]

Developmental Cell, Volume *41*

Supplemental Information

Multi-site Neurogenin3 Phosphorylation

Controls Pancreatic Endocrine Differentiation

Roberta Azzarelli, Christopher Hurley, Magdalena K. Sznurkowska, Steffen Rulands, Laura Hardwick, Ivonne Gamper, Fahad Ali, Laura McCracken, Christopher Hindley, Fiona McDuff, Sonia Nestorowa, Richard Kemp, Kenneth Jones, Berthold Göttgens, Meritxell Huch, Gerard Evan, Benjamin D. Simons, Douglas Winton, and Anna Philpott

INVENTORY OF SUPPLEMENTAL INFORMATION

Supplemental Figures

Figure S1 (related to Figure 1) Ngn3 expression and phosphorylation in the embryonic pancreas.

Figure S2 (related to Figure 3) 6S-A Ngn3 phospho-mutant knock-in animals.

Figure S3 (related to Figure 3) Embryonic analysis of 6S-A Ngn3 phospho-mutant animals.

Figure S4 (related to Figure 3) Adult pancreas analysis of WT and 6S-A Ngn3 animals.

Figure S5 (related to Figure 4) Genome-wide transcriptomic analysis of pancreatic organoids expressing WT and 6S-A Ngn3.

Figure S6 (related to Figure 5) 6S-A Ngn3 shows enhanced binding to and activation of Sst.

Figure S7 (related to Figure 6) Islet expansion in a c-Myc-driven insulinoma model.

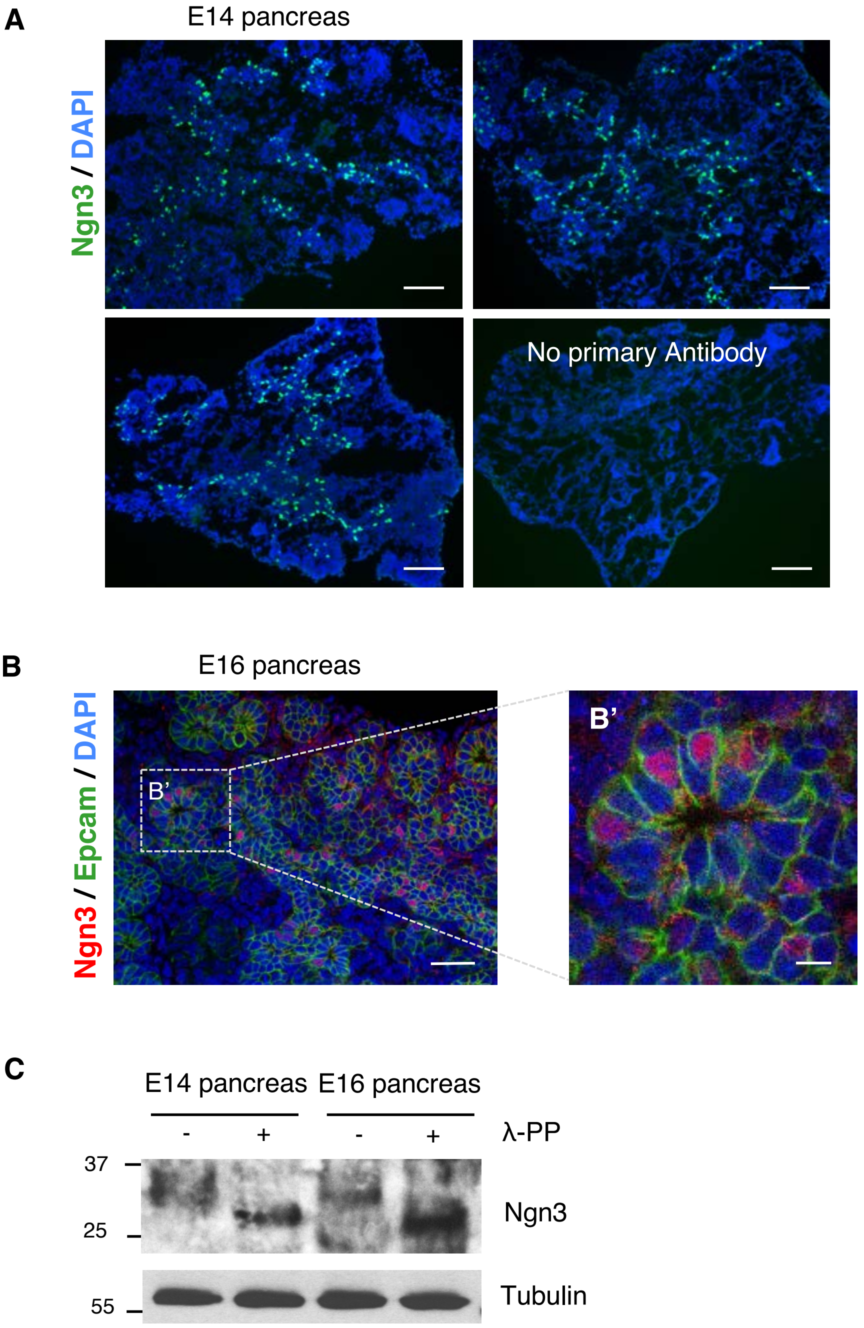

Figure S1 (related to Figure 1) Ngn3 expression and phosphorylation in the embryonic pancreas.
(**A**) Ngn3 immunostaining in E14.5 mouse embryonic pancreas. Section without primary antibody is shown in the bottom right panel; nuclei are counterstained with DAPI (blue). Scale bar: 50 µm. (**B**) Co-staining of Ngn3 (red) and Epcam (green) in E16.5 mouse embryonic pancreas; nuclei are counterstained with DAPI (blue). Scale bars: 50 µm (**B**) and 10 µm (**B'**). (**C**) Additional representative pictures of Western blotting of endogenous Ngn3 protein expression and phosphorylation in the embryonic pancreas (λ-PP = phosphatase λ).

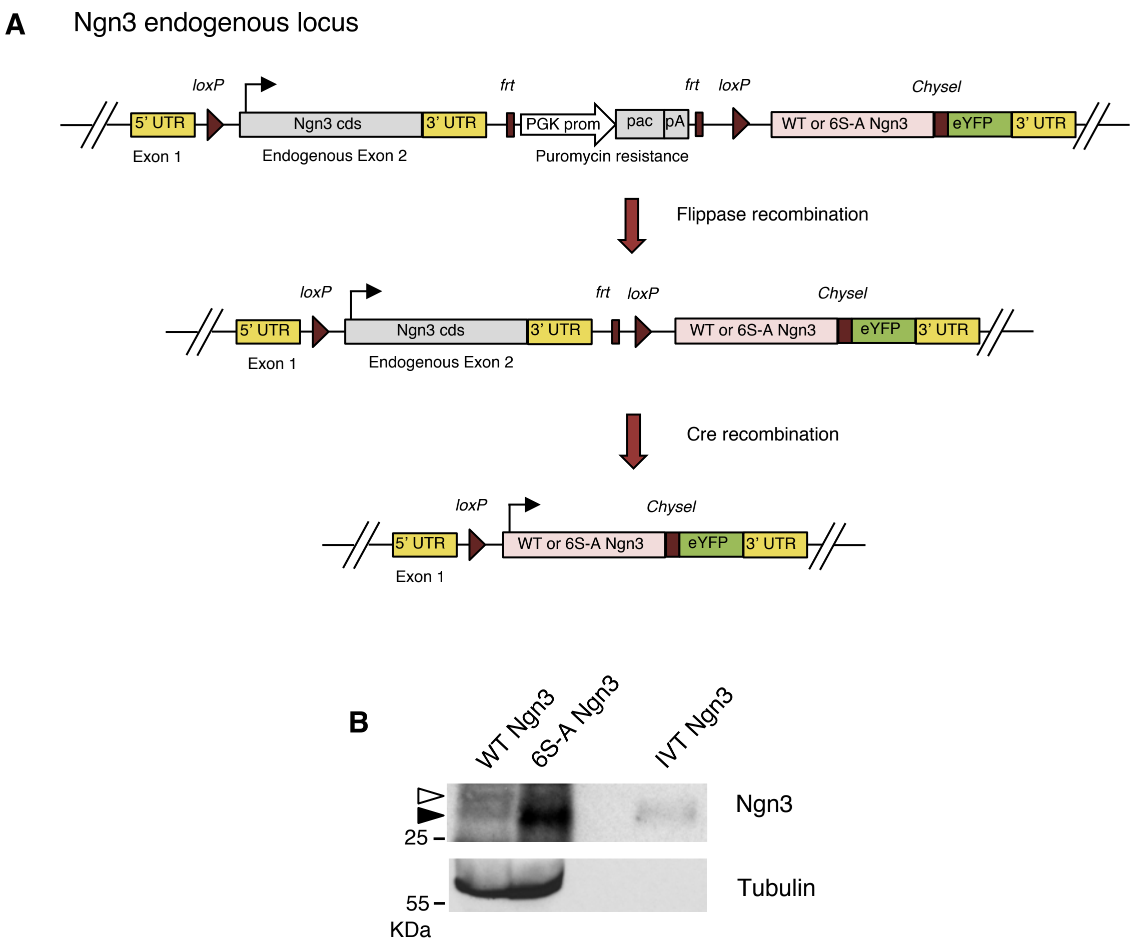

Figure S2 (related to Figure 3) 6S-A Ngn3 phospho-mutant knock-in animals.
(**A**) Schematic diagram of the generation of transgenic animals carrying phosphomutant 6S-A Ngn3 and eYFP or WT Ngn3 and eYFP in place of endogenous Ngn3. The endogenous Ngn3 locus followed by puromycine resistance cassette is flanked by loxP sites and the puromycin resistance cassette is flanked by frt sites. Following Flippase-mediated recombination, the puromycin resistance cassette is removed. Subsequent Cre-mediated recombination replaces endogenous Ngn3 coding sequence with WT Ngn3^{eYFP} or 6S-A Ngn3^{eYFP} alleles. (**B**) Western blot showing endogenous Ngn3 expression in WT and 6S-A mutant animals. This demonstrates that Ngn3 is not phosphorylated on 6 SP sites in 6S-A Ngn3 mutants. Tubulin is used as a loading control. Solid and open arrowheads indicate un(der)phosphorylated and phosphorylated Ngn3, respectively

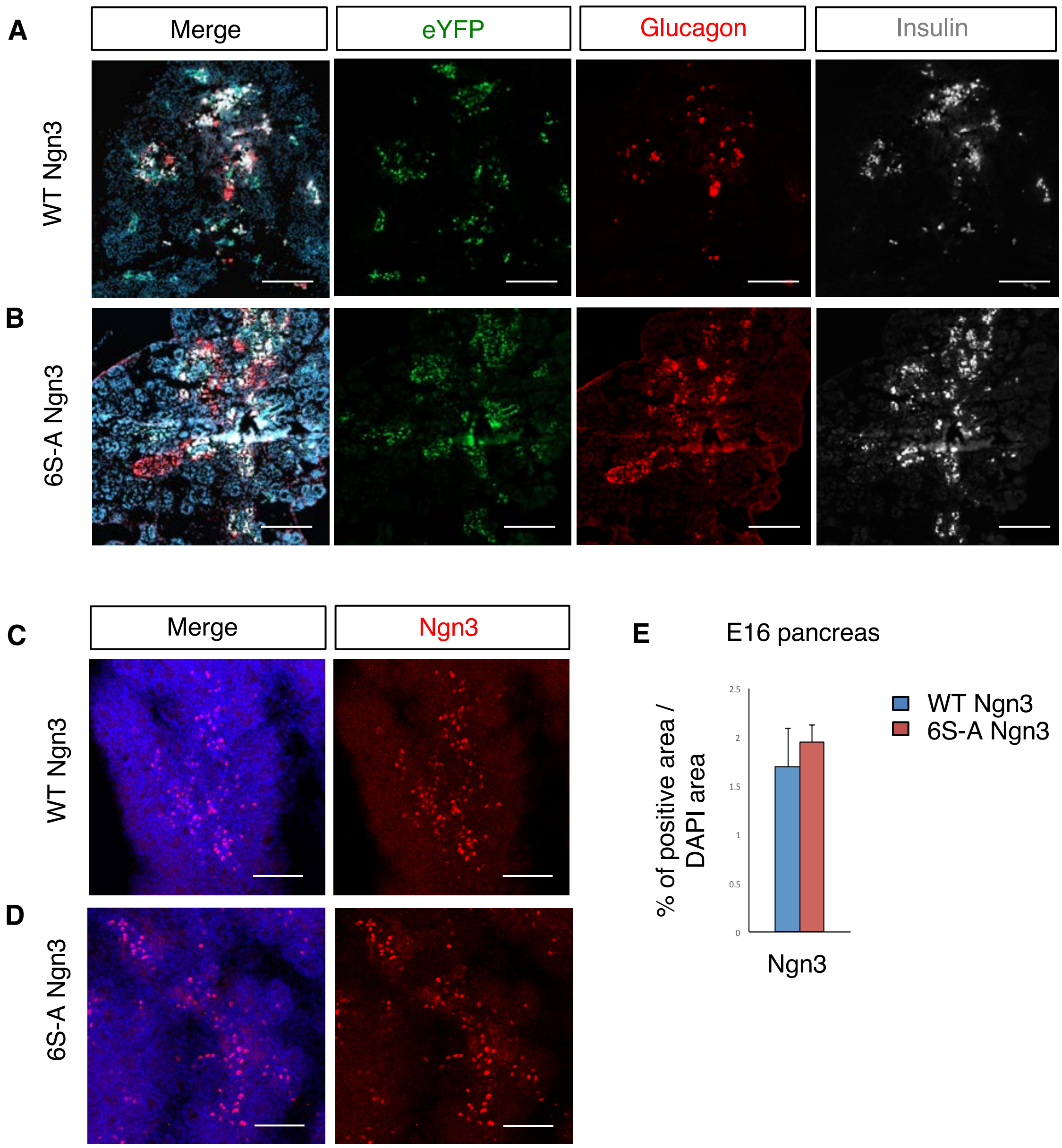

Figure S3 (related to Figure 3) Embryonic analysis of 6S-A Ngn3 phospho-mutant animals.
(**A-B**) Additional representative pictures of immunohistochemistry for eYFP (green), glucagon (red) and insulin (grey) in E16 embryonic pancreas from WT (**A**) and 6S-A (**B**) Ngn3 animals, nuclei counterstained with DAPI (blue). Scale bar: 200 µm. (**C-E**) Immunostaining for Ngn3 (red) and quantification of the % of Ngn3+ cells in E16 embryonic pancreas; nuclei are counterstained with DAPI (blue). Scale bar: 100µm n=3 Mean ± s.e.m.

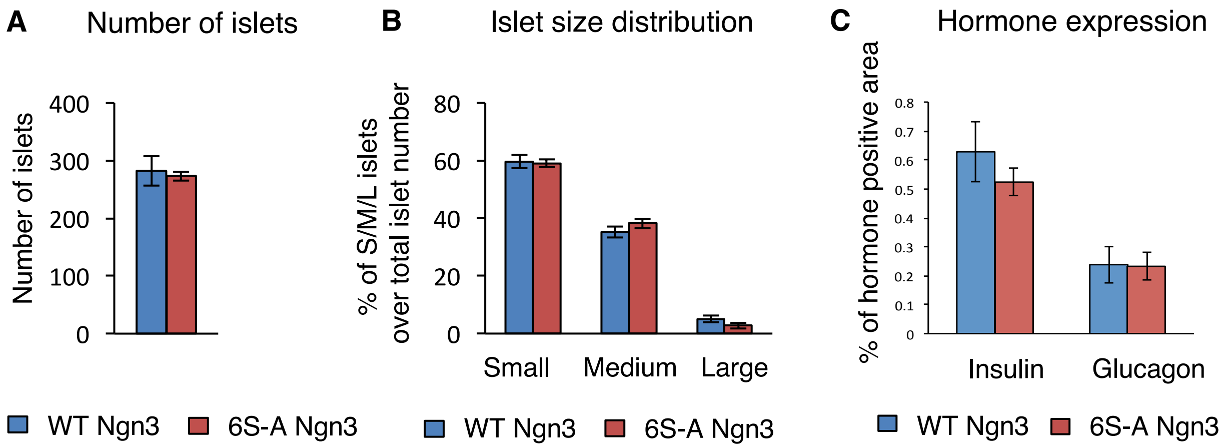

Figure S4 (related to Figure 3) Adult pancreas analysis of WT and 6S-A Ngn3 animals.
(**A-B**) Graphs showing the average total number of islets (**A**) and their size distribution (**B**) in WT and 6S-A adult mouse pancreata. Data represent mean ± s.e.m. n=4 from 6-8 sections for each animal, 100μm apart.
(**C**) Quantification of the % of insulin-positive and glucagon-positive areas in WT Ngn3 and 6S-A Ngn3 adult pancreata. Data represent mean ± s.e.m. n=4 from 6-8 sections for each animal, 100μm apart.

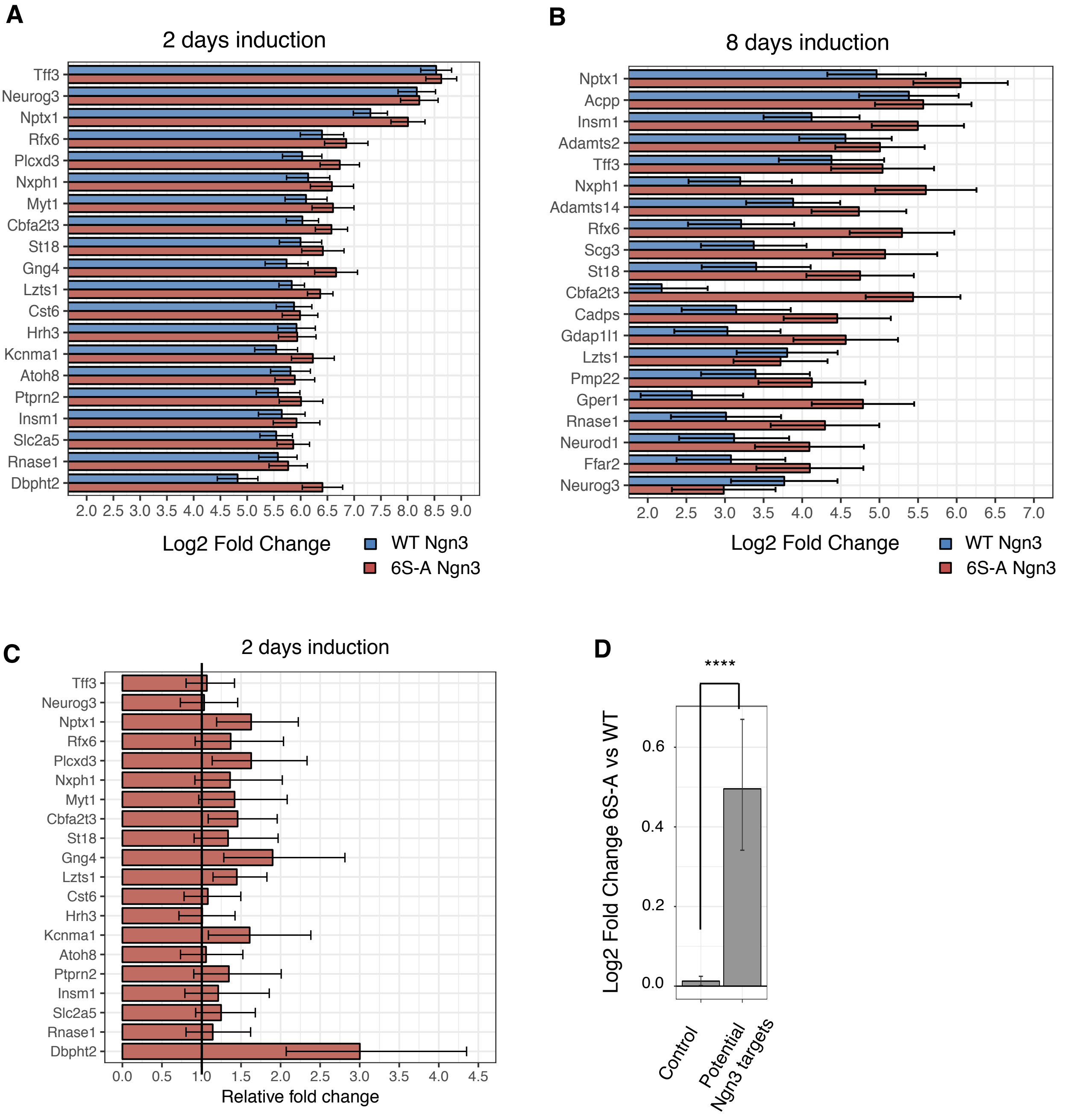

Figure S5 (related to Figure 4) Genome-wide transcriptomic analysis of pancreatic organoids expressing WT and 6S-A Ngn3.

(**A-B**) Graph showing the relative log2 fold change of expression of the top 20 genes upregulated in WT and 6S-A Ngn3 compared to control (GFP only) at 2 (**B**) and 8 (**A**) days. (**C**) Graph showing the relative fold change of gene expression in 6S-A Ngn3 organoids compared to WT Ngn3 fold change (WT Ngn3 set as 1 unit) at 2 days after Ngn3 induction. Data represent log2 fold change ± SEM (n = 3; A, B) and mean fold change ± SEM (n = 3; C). (**D**) Average relative log2 fold change of expression in potential Ngn3 targets compared to control (all genes excluding Ngn3 targets) at 2 days. **** p<0.0001. Data represent average log2 fold change and error bars represent 95% confidence intervals of the mean (n = 3).

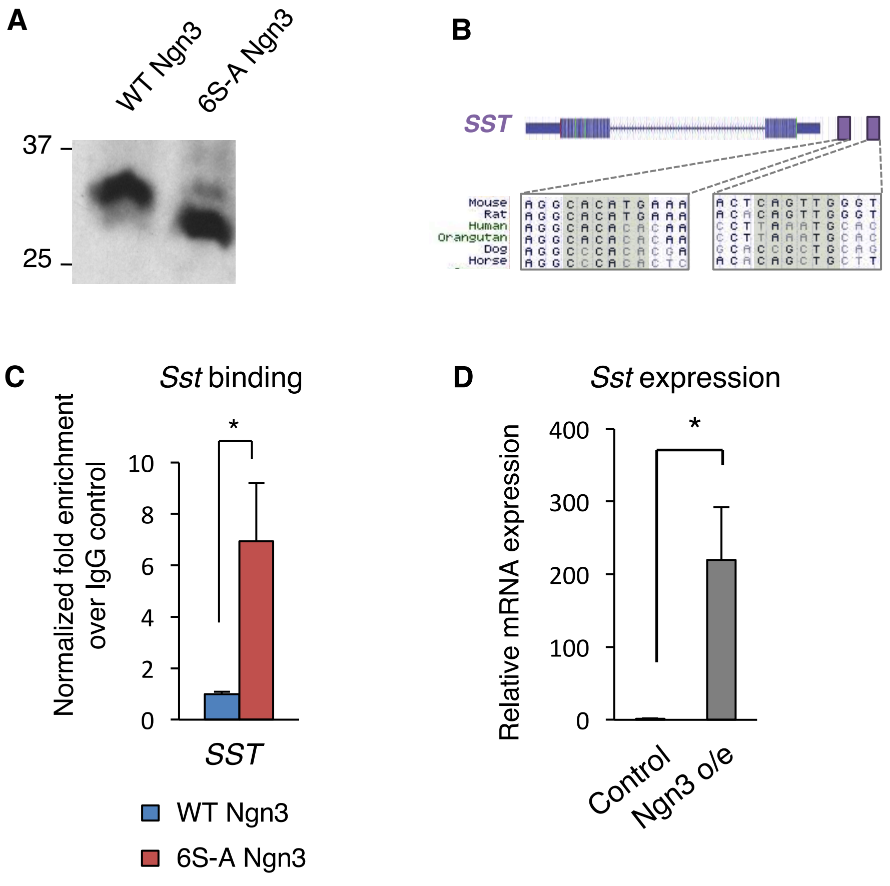

Figure S6 (related to Figure 5) 6S-A Ngn3 shows enhanced binding to and activation of *Sst*.
(A) Western blot for HA-tagged WT and 6S-A Ngn3 on chromatin enriched extracts from transfected mPAC cells before immunoprecipitation. (**B**) Sequence and conservation of 2 E-boxes in *Sst* promoter region (**C**) ChIP analysis of ductal mPAC cell extracts expressing WT and 6S-A Ngn3. Data represent mean ± s.e.m. (n≥3) Student's t-test, * p<0.05. (**D**) Relative mRNA expression of Sst after Ngn3 overexpression in ductal mPAC cells, normalized to β-actin. Data represent mean ± s.e.m (n=3). Student's t-test, * p<0.05

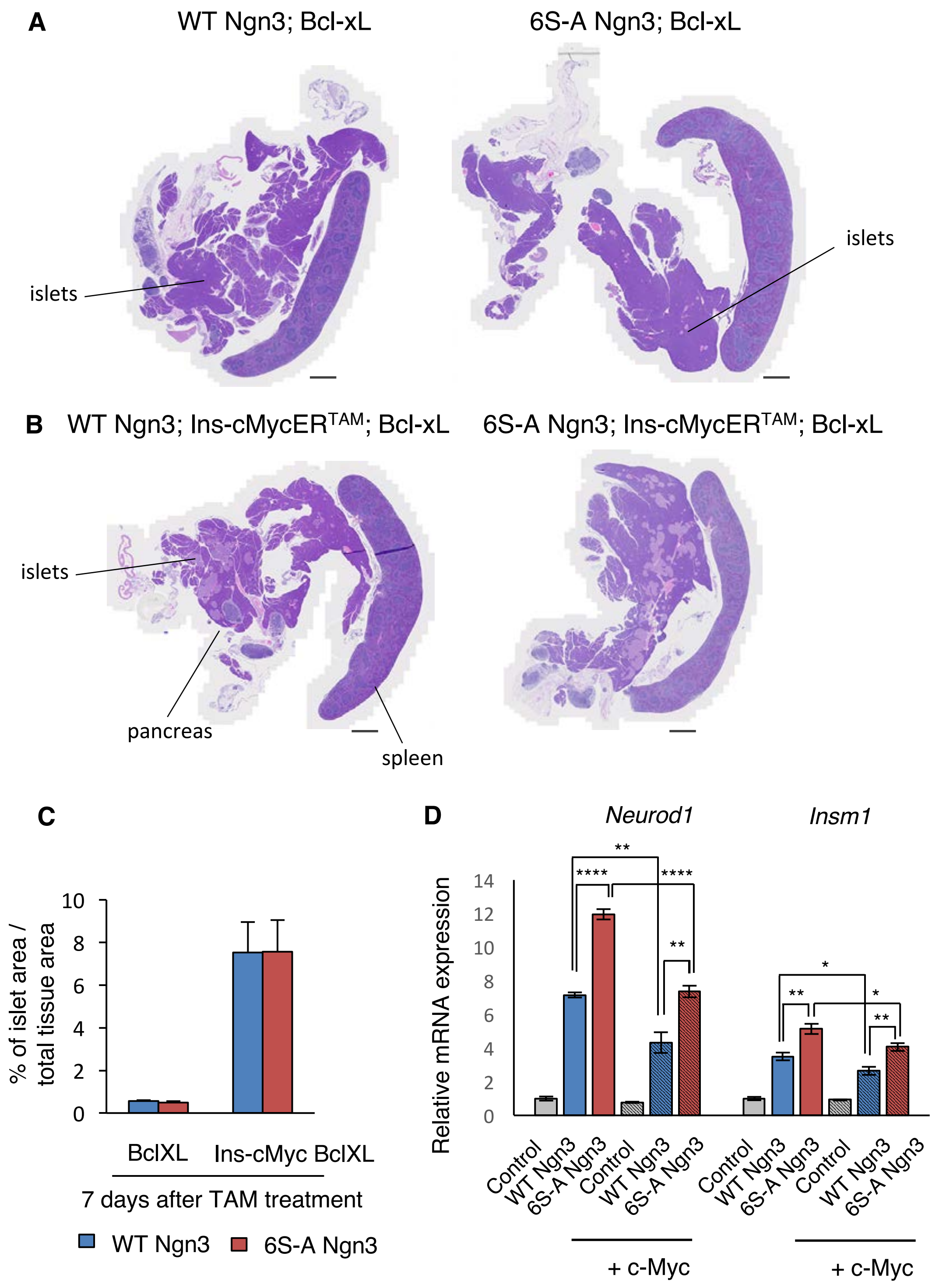

Figure S7 (related to Figure 6) Islet expansion in a c-Myc-driven insulinoma model.

(**A-B**) Haematoxylin and eosin staining of pancreata from WT and 6S-A Ngn3 animals crossed with Bcl-xL only (**A**) or Ins-cMycER^{TAM}; Bcl-xL (**B**), 7 days after tamoxifen treatment. Scale bar: 1000μm (**C**) Quantification of the % of islet area in Bcl-xL and Ins-cMycER^{TAM}; BclXL mice at 7 days post tamoxifen injection. Data is mean ± s.e.m. n≥3 different animals each genotype (2-11 sections each animal). (**D**) Relative mRNA expression of *Neurod1* and *Insm1* in Xenopus embryos expressing WT or 6S-A Ngn3 with or without c-Myc, as indicated. Data is mean ± s.e.m (n=4). Student's t-test, * $p<0.05$, ** $p<0.01$, **** $p<0.0001$
